# Factors affecting fruit and vegetable consumption and purchase behavior of adults in sub-Saharan Africa: A rapid review

**DOI:** 10.3389/fnut.2023.1113013

**Published:** 2023-04-11

**Authors:** Barbara Stadlmayr, Ursula Trübswasser, Stepha McMullin, Alice Karanja, Maria Wurzinger, Laura Hundscheid, Petra Riefler, Stefanie Lemke, Inge D. Brouwer, Isolde Sommer

**Affiliations:** ^1^Institute for Development Research, Department of Sustainable Agricultural Systems, University of Natural Resources and Life Sciences, Vienna, Austria; ^2^World Agroforestry (ICRAF), Nairobi, Kenya; ^3^Division of Human Nutrition and Health, Wageningen University, Wageningen, Netherlands; ^4^Institute for Marketing and Innovation, Department of Economics and Social Science, University of Natural Resources and Life Sciences, Vienna, Austria; ^5^Center for Agroecology, Water and Resilience, Coventry University, Coventry, United Kingdom; ^6^Division of Human Nutrition and Health/CGIAR Initiative Sustainable Healthy Diets (SHiFT), Wageningen University, Wageningen, Netherlands; ^7^Department for Evidence-Based Medicine and Evaluation, University for Continuing Education, Krems, Austria

**Keywords:** food environment, consumer behavior, diets, sub-Saharan Africa, sustainable food systems, fruit, vegetables

## Abstract

In order to achieve the Sustainable Development Goals, considerable dietary shifts, including an increase in the consumption of fruit and vegetables (FV) will be required. However, worldwide consumption of FV is far below international recommendations, including in many low- and middle-income countries (LMICs), particularly in Africa. Understanding what, where, when, and how people choose to eat requires an understanding of how individuals are influenced by factors in their social, physical, and macro-level environments. In order to develop effective interventions to increase fruit and vegetable consumption, the factors influencing consumer behavior need to be better understood. We conducted a rapid review to assess and synthesize data on individual, social, physical, and macro-level factors that enable or constrain fruit and vegetable consumption and purchase among adults living in sub-Saharan Africa. Our conceptual framework is based on a socio-ecological model which has been adapted to settings in LMICs and Africa. We systematically searched four electronic databases including Scopus, Medline (PubMed), PsycInfo, and African Index Medicus, and screened Google Scholar for gray literature. We included a total of 52 studies and narratively summarized the existing evidence for each identified factor across the different levels. We found that most studies assessed demographic factors at the individual level including household or family income, socio-economic status and education. Furthermore we identified a variety of important factors that influence FV consumption, in the social, physical, and macro environment. These include women's empowerment and gender inequalities, the influence of neighborhood and retail food environment such as distance to market and price of FV as well as the importance of natural landscapes including forest areas for FV consumption. This review identified the need to develop and improve indicators both for exposure and outcome variables but also to diversify research approaches.

## Introduction

Dietary patterns are changing worldwide with a general trend toward unhealthy diets (1, 2). Suboptimal diets are key risk factors for all forms of malnutrition, including undernutrition, micronutrient deficiencies, overweight and are among the greatest societal challenges which lead to health, economic and environmental burdens (3, 4). Most low-and middle-income countries (LMICs), particularly in Africa, are experiencing a dietary transition from traditional to highly processed foods, mostly driven by globalization and urbanization (5, 6).

Fruit and vegetables (FV) are rich in vitamins, minerals, phytochemicals and fiber, and are regarded as essential for healthy and sustainable diets (2, 7). Diets that are rich in FV provide promising solutions to micronutrient deficiencies and are associated with a reduced risk of non-communicable diseases such as cardiovascular diseases, diabetes, hypertension, and cancer (4, 8). However, despite the positive benefits of FV, global consumption is far below the WHO recommendation of 400 grams or more FV (equivalent to 5 servings of 80 g each) per day. In LMICs, over 80% of the population consume less than the recommended amounts (7, 9, 10).

What, where, how, and when people choose to eat or acquire food requires an understanding of the multiple influences ranging from a variety of personal and interpersonal factors to more distant, structural issues (11–15). The importance of improving diets through a holistic food systems perspective is widely acknowledged in the literature (14, 16, 17). Within the sustainable food systems framework developed by the High-Level Panel of Experts on Food Security and Nutrition (HLPE), food supply chains, food environments, and consumer behavior are core elements influencing diets (14, 16). Food environments connecting the wider food system with diets have received increasing attention in global policy and research agendas (14, 16) and different conceptual frameworks have been developed for LMICs in recent years (18, 19). They often focus on personal (e.g., affordability, convenience) and external domains (e.g., availability, price, marketing regulations), but less on social aspects including influences through social interactions, social support, gender and social norms, or role modeling (13). For the present review, we therefore followed a socio-ecological model (12) which was adapted for the African context (13, 20). It focuses on the relationship between people and their social (e.g., family, friend, community influence), physical (e.g., access and availability in the neighborhood, at home, in food outlets) and macro-level (e.g., sociocultural norms, agricultural policies) environments in understanding fruit and vegetable consumption and purchase.

Previous systematic reviews in Africa focused either on dietary behavior in urban African environments (20, 21), on dietary and physical activity behaviors in urban sub-Saharan Africa (SSA) (22) or on household economic and demographic determinants of fruit and vegetables (23). Currently, no review has assessed consumption and purchase behavior with regard to FV in sub-Saharan Africa and their multiple factors of influence. This review, therefore, aims to assess and synthesize data at the individual level and at the social, physical, and macro-level environment that affect fruit and vegetable consumption and purchase by adults in sub-Saharan Africa. The findings of our review will identify gaps and help guide future research and policy.

## Methods

### Review typology

To ensure methodological quality, we followed the Cochrane rapid review recommendations (24) and the Preferred Reporting Items for Systematic Reviews and Meta-Analyses (PRISMA) (25). Rapid reviews follow the systematic approach of traditional systematic reviews, but aim to fasten the process to achieve manageable and timely evidence. Restrictions include for example, limiting the publication language to English, limiting the number of outcomes, or date restrictions (24). We drafted a review protocol and registered it a priori on PROSPERO (CRD42021248475 available from https://www.crd.york.ac.uk/prospero/display_record.php?RecordID=248475). Due to resource limitations, we made an amendment to the protocol by excluding experimental studies.

### Conceptual framework

We developed an initial conceptual framework based on a socio-ecological model (12) and its adaptation for Africa (11) to guide our review. The socio-ecological model describes the multiple influences on what people eat at the individual/household level (e.g., biological, demographic lifestyle/behavioral factors), the social level (e.g., influence of family, friend, community), the physical level (e.g., access and availability in the neighborhood, at home, in food outlets), and the macro-level (e.g., sociocultural norms, agricultural policies). In addition, we used two food environment frameworks for LMICs (18, 19) for potential exposure variables such as convenience, food safety, and distance to market and the food systems framework from the High-Level Panel of Experts on Food Security and Nutrition (HLPE), for the outcome variables (16) to inform our initial framework.

The outcome variable “consumer behavior” was adapted from the HLPE framework, which defines consumer behavior as “all the choices and decisions made by consumers, at the household or individual level, on what food to acquire, store, prepare, cook and eat, and on the allocation of food within the household (including gender repartition and feeding of children) (16). In our review, consumer behavior refers to the purchase and consumption of FV in terms of “what,” “how,” “where” and “when” FV is consumed or purchased. “What” includes the quantity of FV consumed or purchased, or if FV were consumed and purchased or not. “How” refers to the frequency of FV consumption and food combinations, and how people interact with the social and physical environment to consume and purchase FV. “Where” refers to the location of FV consumption or purchase, and “When” refers to the timing of consumption or purchase. The adapted framework is presented in **Figure 3** in the Results section.

### Inclusion and exclusion criteria

We used the Population, Exposure, Context, Outcome (PECO) framework to develop the eligibility criteria. We selected articles following these inclusion criteria: (i) Population: healthy adults, men, and women, aged 18–65 years (80% of all participants in the papers falling in this range); (ii) Exposure: individual, social, physical and macro-level factors affecting food and purchase behavior; (iii) Context: all sub-Saharan African countries, rural-urban, peri-urban areas; (iv) Outcome: fruit and vegetable consumption, or purchase behavior at individual level; Study designs eligible for our review were: observational studies including cross-sectional, cohort or case-control study. Only studies published in English between January 2000 to April 2022 were included The timeframe was chosen to include all articles published since WHO recommended to eat 400 g or more FV per day at the beginning of the 2000s (7). Studies were excluded if they addressed non-human or clinical populations, qualitative study design, non-English publications, and were outside of sub-Saharan Africa.

### Literature search

For this review, we systematically searched four electronic databases: Scopus, MEDLINE (PubMed), PsycInfo, and African Index Medicus. For each database, we applied specific indexing terms, such as Medical Subject Headings (MeSH) terms for MEDLINE (PubMed) and free text terms. We developed an initial search syntax for Scopus and thereafter adapted it for the respective databases. In addition, we screened reference lists of relevant reviews to identify relevant articles. We searched Google Scholar for gray literature.

### Screening

We imported all references into the CADIMA platform (https://cadima.info) to check titles and abstracts against inclusion and exclusion criteria and to document the review process. The first author (BS) conducted title and abstract screening with a 40% dual screening of title and abstracts by co-authors (UT, LH, IS, AK, SM). In case of doubt, we included the reference to the next stage. For full-text screening, we transferred included titles and abstracts from CADIMA to Excel. The first author (BS) screened all included full-text articles and co-authors (UT, LH, IS, and AK) double-screened 40% full-texts. Disagreements in selection were resolved through discussion among authors.

### Data extraction

We extracted data by applying a standardized data extraction spreadsheet in Excel. The first author (BS) extracted data from included studies. Co-authors (UT, LH, and AK) checked the correctness and completeness of extracted data (40%). Extracted data included (1) study characteristics: title, author(s), year of publication, country, setting (urban, rural, peri-urban), study design, primary or secondary data; (2) sample characteristic: gender/sex, age (range and/or mean), sample size; (3) exposures: individual, social, physical and macro level factors categorized based on a socio-ecological framework, exposure tool, unit of exposure; (4) outcome: outcome unit, outcome measurement tool; and (5) results: methods of analysis, effect sizes, *p* values.

We were interested in exploring relationships between the exposure/factor and outcome variables assessed by correlation or regression analysis. In addition, we also considered methods that assessed statistically significant differences between groups, e.g., seasonal differences in FV consumption, using t-tests, Wilcoxon signed- rank tests, or ANOVA to include a wide range of factors that are listed separately in the evidence tables. The cut-off for statistical significance was a *p-*value of < 0.05.

### Risk of bias assessment

The risk of bias was assessed alongside the data extraction process using the Appraisal tool for Cross-Sectional Studies (AXIS) (26). For longitudinal studies, we adapted the AXIS tool with questions from a Quality Assessment Tool for Quantitative Studies developed by the Effective Public Health Practice Project (EPHPP) (27). The first author (BS) rated the risk of bias and co-authors (UT, LH, AK) verified 30% of the judgments (Supplementary material 1: Risk of bias assessment). Risk of bias was categorized into high, moderate and low.

### Data synthesis

Due to the heterogeneity of studies and variation in outcome reporting, we performed a narrative synthesis of the findings from the included studies, guided by the levels of our conceptual framework. We categorized the identified factors at the different levels according to the socio-ecological model, as described above. We synthesized FV consumer behavior as (i) consumption or purchase, followed by (ii) fruit and vegetable categories: fruit and vegetables as a separate measure (F, V), combined measure of fruit and vegetables (FV), only fruit (F), or only vegetables (V), and (iii) what, where, when, and how they were purchased or consumed.

## Results

### Characteristics of included studies

**Figure 1:**
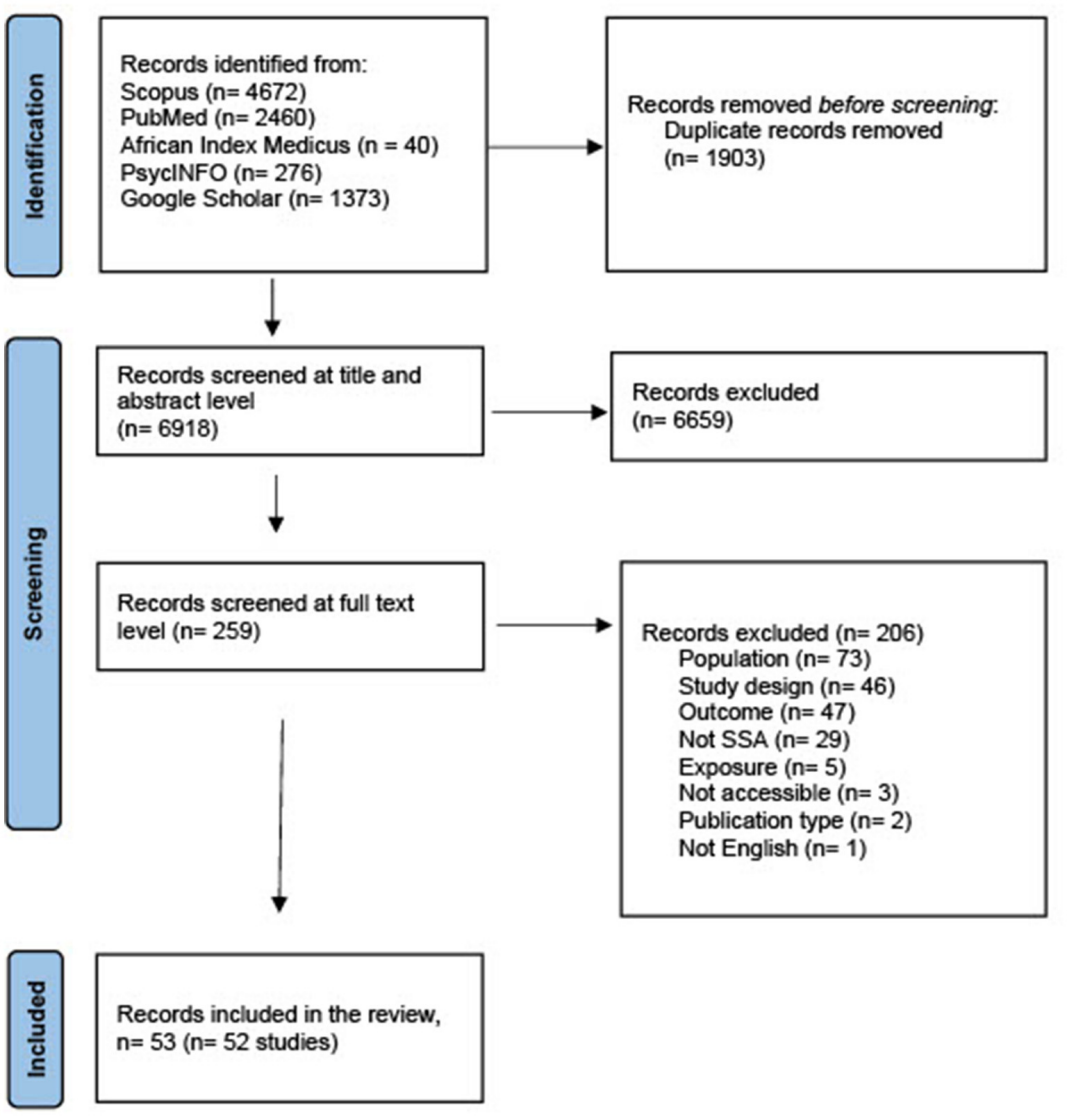
Flow diagram.

The search in four databases and Google scholar identified 8,821 records. After the removal of duplicates, we screened 6,918 records at the title and abstract stage. We identified 259 studies for full-text screening, out of which 52 studies (53 records) met the eligibility criteria and were included in the review. Figure 1 shows the study selection process and related PRISMA flow diagram.

**Figure 2:**
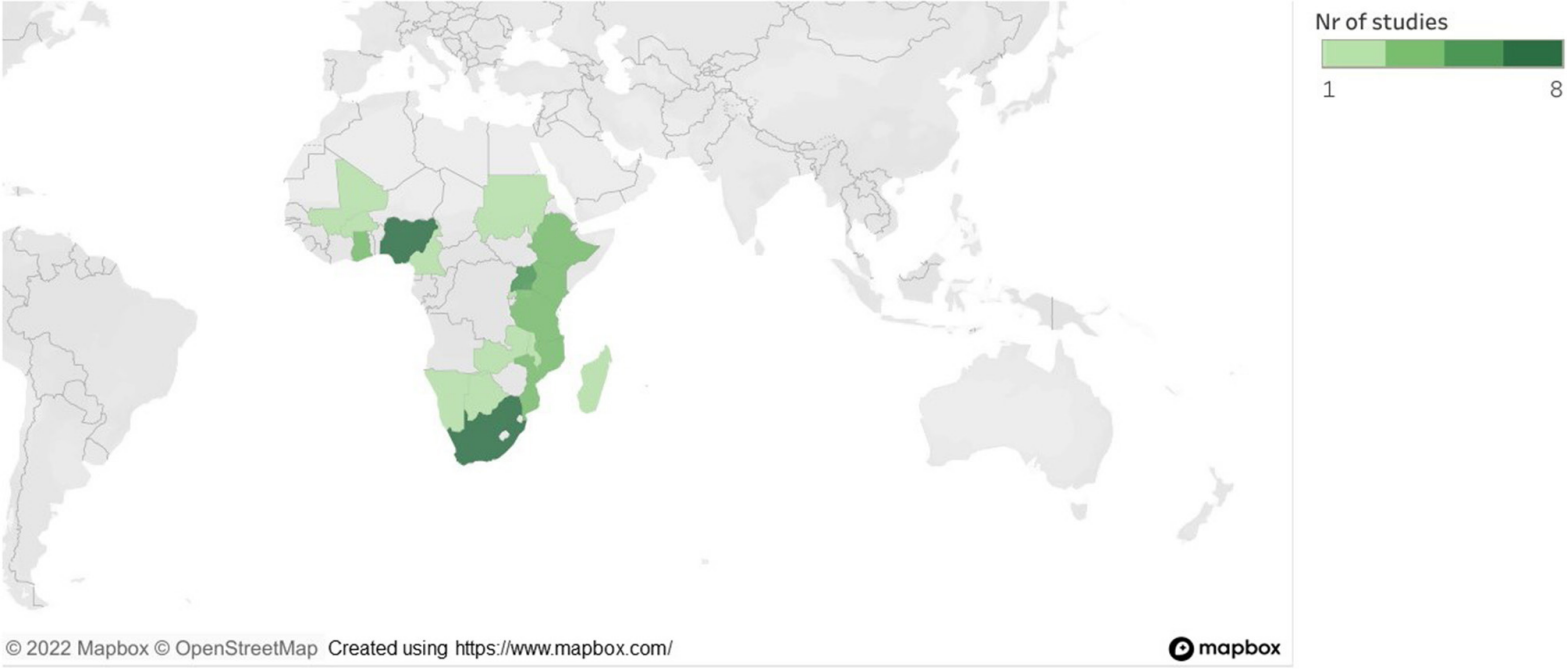
Geographic distribution of included studies across SSA (series: number of studies). Created using https://www.mapbox.com/.

In total, 53 references, representing 52 studies, met the inclusion criteria and were considered in the review. Table 1 provides an overview of the characteristics of the included studies. Figure 2 shows the geographic distribution of included studies across SSA.

**Table 1:** Characteristics of included studies.

**References**	**Risk of bias^1^**	**Country**	**Setting^2^**	**Data source^3^**	**Study design^4^**	**Gender, sex^5^**	**Age in years**	**Sample size**	**Individual**	**Social**	**Physical**	**Macro**	**F, V, FV^6^**	**Consumer behavior**	
														**Consumption, purchase**	**What, how, where, when**
Adenegan et al. (28)	H	Nigeria	U, R	P	CSS	F, M	NR	200	✓				F, V	Purchase	What
Adeoye et al. (29)	H	Nigeria	U	P	CSS	F, M	21–60+	150	✓	✓	✓		V	Purchase	What
Amare et al. (30)	L	Ethiopia	U	P	CSS	F, M	18–65+	356	✓				F, V	Consumption	What, how
Amo-Adjei and Kumi-Kyereme (31)	M	Ghana	U, R	S	CSS	F, M	15–59	9,484	✓	✓		✓	F, V	Consumption	What
Badurally et al. (32)	H	Mauritius	U, R	P	CSS	F, M	NR	374	✓	✓			F, V	Consumption	What
Banwat et al. (33)	H	Nigeria	U	P	CSS	F, M	18–60+	250	✓				FV	Consumption	What
Bhurosy and Jeewon (34)	M	Mauritius	U, R	P	CSS	F	18–65	400	✓				F, V	Consumption	How
Bloomfield et al. (35)	L	Kenya	PU	P	CSS	F, M	16–64 +	4,037	✓				FV	Consumption	What
Bosha et al. (36)	M	Ethiopia	R	P	LONGL	F	20–40	578				✓	FV, F, V	Consumption	What
De Filippo et al. (37)	L	Nigeria	U, PU	P	CSS	F, M	18–65	632	✓	✓	✓		FV	Consumption	What
Demmler et al. (38)	L	Kenya	U	P, S	LONGL	F, M	18 +	1,199			✓		FV	Consumption	What
Gelibo et al. (39)	M	Ethiopia	U, R	P	CSS	F, M	15–69	10,260	✓	✓			FV	Consumption	What
Hall et al. (40)	L	Tanzania	R	S	LONGL	F, M	NR	1,256	✓	✓		✓	FV, F, V	Consumption	What
Jordan et al. (41)	L	Uganda	U, R	P	LONGL	F	30.95	445				✓	FV, F, V	Consumption	What, where
Kabwama et al. (42)	L	Uganda	U, R	S	CSS	F, M	18–69	3,962	✓	✓			FV	Consumption	What
Keding et al. (43)	L	Kenya	R	P	LONGL	F	40.2 (±16.5)	272	✓		✓	✓	FV, F, V	Consumption	What, how, when
Keetile et al. (44)	M	Botswana	U, R	S	CSS	F, M	< 24–65+	1,178	✓				FV	Consumption	What
Kibr (45)	M	Ethiopia	U	P	CSS	F	15–49	423	✓	✓	✓	✓	FV	Consumption	What
Labadarios et al. (46)	M	South Africa	U, R	P	CSS	F, M	16 +	3,287	✓				FV, F, V	Consumption	What
Lagerkvist et al. (47)	M	Ghana	U	P	CSS	F, M	17–60	332			✓		V	Consumption	How^*^, when
Layade et al. (48)	H	Nigeria	U	P	CSS	F, M	15–34	200	✓		✓	✓	FV	Purchase	What
Leyna et al. (49)	M	Tanzania	R	P	CSS	F, M	15-44	1,014	✓				F, V	Consumption	How
Lomira et al. (50)	M	Uganda	U, R	P	CSS	F, M	NR	400	✓	✓			FV	Consumption	What
MacIntyre et al. (51)	L	South Africa	U, R	P	CSS	F, M	15–80	1,751				✓	F, V	Consumption	What
Mayén et al. (52)	L	Seychelles	U, R	S	CSS	F, M	25–64	2,476	✓				FV	Consumption	How
Modibedi et al. (53)	M	South Africa	U	P	CSS	F, M	NR	254	✓	✓			V	Consumption	How
Msambichaka et al. (54)	M	Tanzania	SU	S	CSS	F, M	15–60+	7,953	✓	✓		✓	FV, F, V	Consumption	What, how
Musaiger et al. (55)	M	Sudan	U	P	CSS	F, M	18–30	400	✓				F, V	Consumption	How
Neergheen-Bhujun et al. (56)	M	Mauritius	U, R	P	CSS	F, M	18–65 +	675	✓	✓			V	Consumption	How
Obayelu et al. (57)	H	Nigeria	U	P	CSS	F, M	< 20–50 +	100	✓	✓	✓		F	Purchase	What
Odunitan-Wayas et al.^b^ (58)	M	South Africa	U	P	CSS	F, M	≥ 18	422			✓		FV	Purchase	How
Odunitan-Wayas et al.^b^ (59)	M	South Africa	U	P	CSS	F, M	18–55+	395	✓		✓		F, V	Purchase	What
Okop et al. (60)	M	South Africa	U, R	P	CSS	F, M	30–75	535	✓		✓		FV	Consumption	What
Onah et al. (61)	M	Uganda, Rwanda, Malawi, Zambia, Mozambique	R	S	CSS	F	28.95	10,041		✓			FV, V	Consumption	What
Oyedele et al. (62)	H	Nigeria	U	P	CSS	F, M	36.7 ± 9.2	311	✓	✓			V	Purchase	What
Padrão et al. (63)	L	Mozambique	U, R	P	CSS	F, M	25–64	12,902	✓				F, V	Consumption	How
Padrão et al. (64)	L	Mozambique	U, R	P	CSS	F, M	25–64	3,298	✓			✓	F, V	Consumption	What
Peltzer and Pengpid (65)	L	South Africa	U, R	S	CSS	F, M	15+	15,310	✓				F, V	Consumption	What
Peltzer and Promtussananon (66)	M	South Africa	R, PU	P	CSS	F, M	18–64	200	✓				FV	Consumption	What
Pengpid and Peltzer (67)	M	Kenya	U, R	S	CSS	F, M	18–69	4,479	✓				FV, F, V	Consumption	What
Raaijmakers et al. (68)	M	Nigeria	U	P	CSS	F	18–55	1,220	✓		✓		V	Consumption	What, how
Ravaoarisoa et al. (69)	L	Madagascar	R	P	LONGL	F	18–45	608				✓	F, V	Consumption	How
Reyes-García et al. (70)	L	Cameroon	R	P	LONGL	F, M	16+	160				✓	FV, F, V	Consumption, Acquisition	What, where
Riha et al. (71)	L	Uganda	R	P, S	CSS	F, M	13+	7,340				✓	FV	Consumption	What
Savy et al. (72)	L	Burkina Faso	R	P	LONGL	F	<20–30+	550				✓	FV, F, V	Consumption	What
Sinyolo et al. (73)	M	South Africa	U, R	P, S	CSS	F, M	45.72	20,908	✓	✓	✓		F, V	Consumption	What, how
Subratty and Jowaheer (74)	H	Mauritius	U, R	P	CSS	F, M	15–60	1,213	✓				F	Consumption	How, when
Tata et al. (75)	M	Cameroon	R	P, S	CSS	F	29.7 ± 7.032	247				✓	FV, F, V	Consumption	What
Thakwalakwa et al. (76)	L	Malawi	U, R	P	LONGL	F	27.8 ± 6.0	274	✓	✓	✓	✓	F, V	Consumption	What, when
Torheim et al. (77)	M	Mali	R	P	CSS	F, M	15–45	491	✓				F	Consumption	What
Unwin et al. (78)	M	Tanzania	U, R	P	LONGL	F, M	15-59	209				✓	F, V	Consumption	What
Wang et al. (79)	H	Ghana	U	P	CSS	F, M	39.2	1,100	✓	✓			F	Consumption	How
Yaya and Bishwajit (80)	M	Namibia	U, R	S	CSS	F, M	15–49	14,185	✓	✓	✓		FV	Consumption	What

1 Risk of bias: H, high; M, moderate; L, low;

2 Setting: R, rural; U, urban; PU, peri-urban; SU, semi-urban;

3 Data source: P, primary; S, secondary;

4 Study design: CSS, Cross-sectional study; LONGL, longitudinal study;

4 Gender, sex: F, female; M, male;

6 F;V;FV; F, Fruit; V, Vegetable; FV, Fruit and vegetables combined;

a,b References referring to one study;

How^*^: referring to how foods were treated before consumption.

The majority of the observational studies adopted a cross-sectional design (81%) and the remaining studies (19%) adopted a longitudinal study design, out of which two were panel studies with time intervals of several years. Most studies (73%) collected original data (primary studies), followed by studies that were based on secondary data (17%), or on both primary and secondary data (10%).

Most of the studies included adult women and men (79%). While fewer studies (21%) focused solely on women, and no study looked at the fruit and vegetable consumer behavior of only men. Population characteristics across the studies were heterogeneous and included women of reproductive age, supermarket shoppers, university students, low-income urban residents, adults in rural areas, adults in resource-poor communities, consumers that purchased fresh vegetables at open-air markets.

Fruit and vegetables were mainly assessed at the food group level (83%) and only a few assessed single food items (17%). The outcome variables were presented in the studies either as separate measures (F, V), as a combined measure (FV), or separately and combined (F, V, and FV) (31%, 29%, and 21%, respectively). Only a few studies focused only on vegetables (V) or only on fruit (F) (12% and 8%, respectively). It was often unclear what was counted as fruit or vegetable, e.g., some studies included potatoes within the vegetable category. As fruit and vegetables were often assessed in combination, it was not possible to systematically distinguish whether fruit or vegetable consumption may be linked to different factors.

Fruit and vegetable consumption in terms of quantity (what) and frequency (how) were the dominant outcome measures. “What” was expressed in various units including grams, portion sizes, number of servings, adequate or inadequate consumption, or percentage of adults that consumed FV. “How” FV were consumed referred mostly to the frequency of consumption expressed either as daily or weekly FV consumption, reduction in the frequency of FV consumed per week and many more.

The most frequently applied measures were self-reported semi-quantitative food frequency questionnaires (FFQ), that assessed consumption frequency and also the portion sizes with showcards or photographs. Several studies used qualitative 24 h recall assessing whether adults consumed FV food groups the previous day or not, while only few studies applied quantitative 24 h recall tools assessing the actual intake (see Supplementary material 2). Few studies focused on the purchase or acquisition of fruit and vegetables (11%). Moreover, few studies were found to assess consumer behavior, other than dietary intakes, such as “when,” referring to the timing or “where,” referring to the location of FV consumption or purchase.

Out of the 48 countries in sub-Saharan Africa, literature from 20 countries was available for inclusion in this review (see Figure 2). The majority of the included studies were conducted in South Africa (*n* = 9) followed by Nigeria (*n* = 8), and Uganda (*n* = 5). Figure 2 shows the geographic distribution of included studies across SSA.

### Risk of bias assessment

Out of the 52 studies included in this review, most studies showed moderate risk of bias (*n* = 25), followed by low risk (*n* = 18) and high risk (*n* = 9). The main weaknesses in several studies was that the sampling frame was not representative of the target population. For example, the target population was referring to adults from a certain geographic region, but the sampling frame was restricted to adults living in one selected town in that region. In addition, several studies did not describe the selection process well. For example, while it was often stated that random sampling was conducted, only a few studies described the sampling in detail or provided information on situational aspects such as how and in what frequency respondents were contacted. Exposure and outcome variables were also poorly described, as information on validated measures were often not mentioned or described superficially. The full risk of bias assessment is provided in the Supplementary material 1: Risk of bias assessment.

### Socio-ecological factors affecting fruit and vegetable consumption and purchase

**Figure 3:**
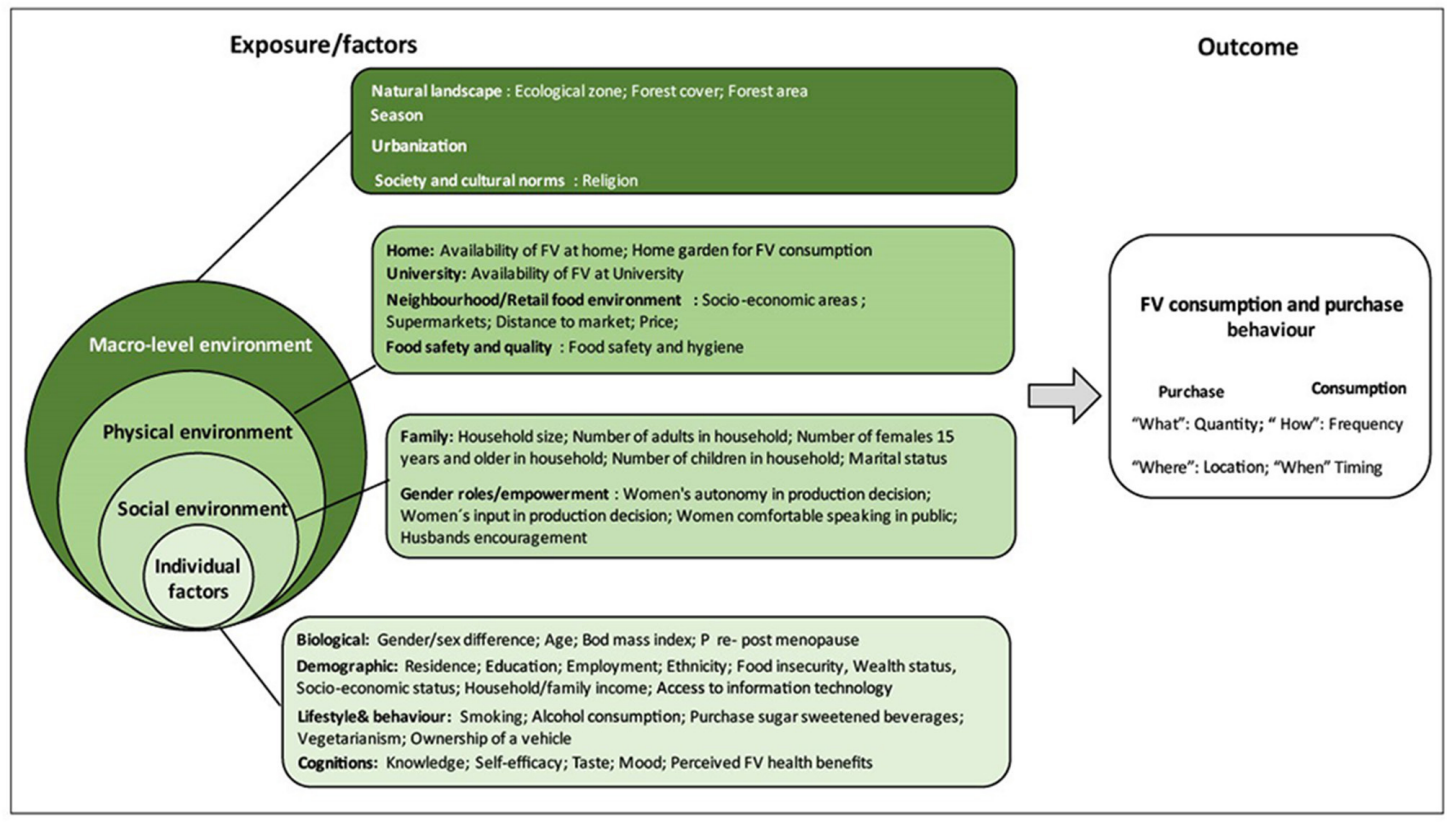
Conceptual framework based on a socio-ecological framework (12) and its adaptation for LMICs (18, 19) and Africa (11) illustrating identified factors in the review that affect fruit and vegetable consumption and purchase. Exposure/factors represented showed an association with the outcome variable or significant differences in the outcome variable. (ii) Outcome: Most outcome variables refers to FV consumption in terms of quantity (“What”) and frequency (“How”).

In this section we present the identified factors, categorized in line with the previously described conceptual framework, and their relevance for fruit, vegetable, and combined FV consumption or purchase among adults in sub-Saharan Africa. Results are presented narratively for each factor. Tables 2–5 provide an overview of the evidence, and Figure 3 illustrates factors that significantly affected FV consumption or purchase among adults in SSA. We adapted our initial conceptual framework by adding new exposure variables/factors that we identified in the literature. Furthermore, we adapted sub-levels within the social, physical, and macro-levels according to the results of our data, after discussion among the review authors. For example, we added the sub-level “Gender roles/empowerment” to the social environment level. In the macro-level environment, we added the sub-level “Natural landscapes,” where we categorized the factors “Ecological zones” and “Forest cover.” The framework shows the diversity of factors across the different levels of influence which highlight the need for multiple, context-specific approaches to improve FV consumption.

**Table 2A:** Individual/household level factors—biological.

**Sub-level**	**Factor**	**Consumer behavior**					Evidence^*^ (References)			
		**Consumption**	**Purchase/acquired**	**What**	**How**	**When**	**Positive association**	**Negative association**	**Significant difference**	**No association/no significant difference**
Biological	Gender/sex (women vs. men)	F		x				(40)	(30)	(32, 40, 46, 64, 67, 73)
		F			x		(54)			(55, 73)
			F	x				(28)		(28, 57)
		V		x			(40, 64, 67)			(30, 32, 40, 46, 64, 73)
		V			x		(54)			(53, 55, 73)
			V	x				(28)		(28, 29, 62)
		FV		x			(39, 52, 54)	(35)	(33, 46)	(40, 42, 50, 60)
			FV	x				(48)		
	Age	F		x			(64)	(40)		(31, 32, 40, 46, 64, 67, 73)
		F			x		(54)	(43)	(74)	(73)
		F				x			(74)	
			F	x						(28, 57)
		V		x			(31, 64, 67, 73)		(46)	(32, 40, 64)
		V			x		(53, 54, 73)		(56)	
			V	x						(29, 62)
		FV		x			(35, 50, 60)	(40)	(46)	(35, 42, 50, 67)
		FV			x		(52, 54)			
	Body mass index (BMI)	F		x						(67)
		F			x		(30)			
		V		x						(67)
		V			x		(30)			
		FV		x						(42, 67)
	Pre-post menopause	F			x				(34)	
		V			x				(34)	

* Evidence: Positive or negative association: Relationship for positive or negative association qualified as statistically significant at the 5% level, based on correlation and regression analysis. Significant differences: tested e.g.„ via t-tests; ANOVA as statistically significant at the 5% level; No association, no significant difference: not statistically significant, or no association. F, Fruit; FV, combined Fruit and Vegetables; V, Vegetables; What: Quantities consumed, amount spent for purchasing FV; % of people consuming F, V, FV; How: Frequency of consumption or purchase; When: referring to the timing of FV consumption.

**Table 2B:** Individual/household level factors—demographic.

**Sub-level**	**Factor**	**Consumer behavior**					Evidence^*^ (References)			
		**Consumption**	**Purchase**	**What**	**How**	**When**	**Positive association**	**Negative association**	**Significant difference**	**No association/no significant difference**
Demographic	Residence (urban vs. rural)	F		x				(31, 64)	(46)	(31, 32, 64, 67, 73)
		F		x		x				(76)
		F			x					(73)
		V		x				(64, 67, 73)	(46)	(31, 32, 64)
		V			x			(73)	(56)	
		FV		x				(67)		(42, 46, 60, 80)
	Education	F		x			(31, 64, 67, 73)		(32)	(40)
		F		x		x				(76)
		F			x		(30, 43, 54, 73, 79)	(79)		
		V		x			(31, 40)	(64)	(32)	(31, 40, 64, 67, 73)
		V			x		(30, 53, 73)		(56)	(54, 73)
			V	x			(62)			(29)
		FV		x			(50, 54, 80)	(35)		(35, 40, 42, 52, 67, 80)
	Employment/occupation	F		x			(31, 43, 73)	(73)		(31, 32, 73)
		F			x		(54, 73, 79)	(73, 79)		(54, 73)
			F	x						(28, 57)
		V		x			(31, 73)	(73)		(31, 32, 73)
		V			x		(73)	(53, 73)	(54)	(54, 56, 73)
			V	x				(28)		(28)
		FV		x			(39, 50, 54)	(54)		(42, 54, 80)
	Ethnicity	F		x			(31, 67, 73)	(31, 73)	(32, 46)	(31, 73)
		F			x		(54, 73)	(73)		(54, 73)
		V		x			(31, 67, 73)	(31, 73)	(32, 46)	(31, 67)
		V			x		(54, 73)	(73)		(54)
		FV		x			(54, 67)		(46)	(54, 67)
	Food insecurity	F		x				(43)	(32)	
		F		x		x		(76)		(76)
		F			x			(49)		
			F	x						(59)
		V		x					(32)	
		V		x		x				(76)
		V			x			(49)		
			V	x						(59)
	Socio-economic status	F		x					(46, 77)	
		F			x				(34)	
		V		x					(46, 68)	
		V			x				(34)	(34)
		FV		x					(46)	
	Wealth status (high vs. low)	F		x			(31, 43, 67, 73)			(31, 40)
		F			x		(73)			
		V		x			(73)	(31)		(31, 40, 67)
		FV		x			(44, 80)			(40, 67)
	Income (family income, household income, parents income, having money)	F		x			(64, 73)		(32)	(64)
		F			x		(30, 73, 79)	(79)		
			F	x						(57)
		V		x			(73)	(64)	(32)	(64)
		V			x		(30, 73)		(56)	
			V	x						(29, 62)
		FV		x			(39)			(37, 60)
		FV			x		(52)			
			FV	x			(48)			

* Evidence: Positive or negative association: Relationship for positive or negative association qualified as statistically significant at the 5% level, based on correlation and regression analysis. Significant differences: tested e.g.„ via t-tests; ANOVA; No association, no significant difference: not statistically significant, or no association. F, Fruit; FV, Fruit and vegetables combined; V, Vegetables; What: Quantities consumed, amount spent for purchasing FV; % of people consuming F, V or FV; How: Represents the frequency of consumption or purchase; When: in reference 76 is referring to season (dry vs. rainy).

**Table 2C:** Individual/household level factors—lifestyle.

**Sub-level**	**Factor**	**Consumer behavior**				Evidence^*^ (References)			
		**Consumption**	**Purchase**	**What**	**How**	**Positive association**	**Negative association**	**Significant difference**	**No association/no significant difference**
Lifestyle	Tobacco use/smoking	F		x			(65)		(32, 65, 67)
		F			x	(63)	(63)		(54, 63)
		V		x					(32, 65, 67)
		V			x	(63)	(63)		(54, 63)
		FV		x					(54, 67)
	Alcohol consumption/drinking habits	F		x					(32, 67)
		F			x		(54)		
		V		x					(32, 67)
		V			x		(54)		
		FV		x			(54)		(67)
	Convenience	V		x					(68)
		FV		x					(45)
	Time	FV		x					(37, 66)
	Physical activity	F		x					(67)
		V		x					(67)
		FV		x					(67)
	Purchased sugar-sweetened beverages	FV		x			(60)		
	Vegetarianism	F		x					(32)
		V		x					(32)
		V			x			(56)	
	Eating out	FV		x					(66)
	Buy FV daily or weekly	FV		x					(60)
	Ownership of a vehicle, Travel to purchase groceries, Ease of transportation	F		x		(73)			
		F			x	(73)			
		V		x		(73)			
		V			x				(73)
		FV		x		(60)			(60)
	Access to information technology (internet, radio, nr. of mobile phones)	F		x		(73)			(73)
		V		x		(73)			
		F			x	(73)			(73)
		V			x	(73)			
	Exposure to media—reading newspapers, magazines	F		x		(31)			
		V		x		(31)			(31)
	Exposure to media—listening to radio	F		x		(31)			(31)
		V		x					(31)
	Exposure to media—watching television	F		x		(31)			(31)
		V		x					(31)

* Evidence: Positive or negative association: Relationship for positive or negative association qualified as statistically significant at the 5% level, based on correlation and regression analysis. Significant differences: tested e.g.„ via t-tests; ANOVA; No association, no significant difference: not statistically significant, or no association. F, Fruit; FV, Fruit and vegetables combined; V, Vegetables; What: Quantities consumed, amount spent for purchasing FV; % of people consuming F, V or FV; How: Represents the frequency of consumption or purchase.

**Table 2D:** Individual/household level factors—Cognition.

**Sub-level**	**Factor**	**Consumer behavior**					Evidence^*^ (References)			
		**Consumption**	**Purchase**	**What**	**How**	**When**	**Positive association**	**Negative association**	**Significant difference**	**No association/no significant difference**
Cognition	Knowledge	V		x			(68)			
		V			x				(56)	
		FV		x						(37, 50, 66)
	Attitude toward FV consumption	FV					(50)			
	Nutrition education	F		x						(32)
		V		x						(32)
		FV		x			(50)			(50)
	Self-efficacy	V		x			(68)			
	Good heating habits (perceived)	FV		x					(66)	
	Food choice motive “health”	V		x			(68)			
	Perceived FV health benefits	FV		x						(37, 45, 60, 66)
	Personal preference	FV		x						(37, 45)
	Mothers preference and perceptions of healthy body size	F		x		x				(76)
	Taste	V		x		x	(76)			
		FV		x						(37)
			FV	x						(48)
	Ethical concern	V		x						(68)
	Mood	V		x			(68)			
		FV		x			(45)			
	Familiar	V		x						(68)

* Evidence: Positive or negative association: Relationship for positive or negative association qualified as statistically significant at the 5% level, based on correlation and regression analysis. Significant differences: tested e.g., via t-tests; ANOVA; No association, no significant difference: not statistically significant, or no association. F, Fruit; FV, Fruit and vegetables combined; V, Vegetables; What: Quantities consumed, amount spent for purchasing FV; % of people consuming F, V or FV; How: Represents the frequency of consumption or purchase; When: in 76 is referring to season (dry vs. rainy).

**Table 3:** Social environment.

**Sub-level**	**Factor**	**Consumer behavior**					Evidence^*^ (References)			
		**Consumption**	**Purchase**	**What**	**How**	**When**	**Positive association**	**Negative association**	**Significant difference**	**No association/no significant difference**
Family	Household size	F			x			(73)		
		F		x			(40)	(40, 73, 79)		(32, 40)
		V			x					(53, 73)
		V		x				(40, 73)		(32)
		V		x	x			(40)		
		FV		x				(40, 50)		(50)
			V	x						(29)
	Number of adults in household	F		x				(79)		
	Number of females 15 years or older in household	F		x						(73)
		F			x		(73)			
		V		x			(73)			
		V			x					(73)
	Number of children in household	F		x			(73)	(79)		
		F			x		(73)			
		V		x	x					(73)
		V		x		x				(76)
	Marital status	F		x						(32)
		F			x		(54)			(31)
			F	x						(57)
		V			x		(54)		(56)	
		V		x						(32)
			V	x						(31, 62)
		FV		x			(39, 42)	(50)		(50, 54)
	Help with procurement and preparation	FV		x						(37)
	Family preferences and habits	FV		x						(37)
	Purchase special foods for children	F		x		x				(76)
		V		x		x				(76)
	Who purchases food within the family (mother; husband; both; other family member)	F		x		x				(76)
		V		x		x				(76)
Gender roles/empowerment	Influence of husband/husband encouragement	FV		x			(45)			
	Woman decides on how family income is used	FV		x						(50)
	Woman decides on type of food eaten in the household	FV		x						(50)
	Women's autonomy in production decision	V		x			(61)			
		FV		x			(61)			(61)
	Women's Input in production decision	V		x			(61)			
		FV		x			(61)			
	Women comfortable speaking in public	V		x						(61)
		FV		x			(61)			

* Evidence: Relationships for positive or negative associations qualified as statistically significant at the 5% level. Relationship for positive or negative association qualified as statistically significant at the 5% level, based on correlation and regression analysis. Significant differences: tested e.g., via t-tests; ANOVA; No association, no significant difference: not statistically significant, or no association. F, Fruit; FV, Fruit and vegetables combined; V, Vegetables; What: Quantities consumed, amount spent for purchasing FV; % of people consuming F, V or FV; How: Represents the frequency of consumption or purchase; When: is referring to seasonal difference (dry vs. rainy) in 76.

**Table 4:** Physical environment.

**Sub-level**	**Factor**	**Consumer behavior**					Evidence^*^ (References)			
		**Consumption**	**Purchase**	**What**	**How**	**When**	**Positive association**	**Negative association**	**Significant difference**	**No association/no significant difference**
Home	Availability of FV at home	FV		x			(45)			
	Home garden for FV consumption/own production of FV	V		x			(73)			
		V			x		(73)			
		F		x						(73)
		F			x					(73)
		FV		x						(37)
	Storage of FV at home	FV		x						(37)
University	Availability of FV at university		F	x			(57)			
			FV	x				(48)		
Neighborhood/retail food environment	Socio-economic areas		F	x	x				(58, 59)	
			V	x	x				(58, 59)	
	Availability of FV in the neighborhood	FV		x						(37)
	Supermarket vs. traditional retail outlets	FV		x				(38)		
	Distance to market	F			x			(43)		
		F		x		x				(76)
		FV		x						(37)
	Price	V								(68)
		FV		x					(37)	(45)
Product property and food safety	Poor product quality	FV		x						(37)
	Size of vegetable item		V	x						(29)
	Type/variety of vegetable item		V	x						(29)
	Food safety and hygiene	V			x	x	(47)	(47)		(47)

* Evidence: Relationship for positive or negative association qualified as statistically significant at the 5% level, based on correlation and regression analysis. Significant differences: tested e.g., via t-tests; ANOVA; No association, no significant difference: not statistically significant, or no association. F, Fruit; FV, Fruit and vegetables combined; V, Vegetables What: Quantities consumed, amount spent for purchasing FV; % of people consuming F, V or FV; How: Represents the frequency of consumption or purchase and how V were prepared at home in case of 47.When: is referring to the timing, i.e. delay in V consumption in 47 and to seasonal differences in 7.

**Table 5:** Macro environment.

**Sub-level**	**Factor**	**Consumer behavior**					Evidence^*^ (References)			
		**Consumption**	**Purchase**	**What**	**How**	**When**	**Positive association**	**Negative association**	**Significant difference**	**No association/no significant difference**
Natural landscape	Ecological zone (forest vs. coastal)	F			x		(31)			
		V			x		(31)			
	Ecological zone (Savannah vs. coastal)	F			x			(31)		
		V			x			(31)		(31)
	Forest cover	F		x			(40)			(40)
		V		x			(40)			(40)
		FV		x			(40)			
	Forest vs. non-forest area	F		x						(75)
		V		x					(75)	(75)
		FV		x					(75)	
Season	Season	F		x				(40)	(43, 72, 76)	(36, 40, 72)
		F		x		x			(41)	(41)
		F			x				(69)	
		V		x					(36, 43, 70, 72, 76)	
		V			x				(69)	(40)
		V		x		x			(41)	(41)
			V			X			(70)	
		FV		x					(43, 70)	(36, 40, 70, 72)
		FV		x		x			(41)	(41)
			FV			x			(70)	
Urbanization	Strata of urbanization	F		x					(51)	
		V		x					(51)	
	Urbanicity level (various levels compared to least urban)	FV		x				(71)		
	Rural to urban migration	FV		x					(78)	(78)
Societal and cultural norms	Religion	F		x				(31)		(31, 54)
		V		x			(31)	(31)		(31, 54)
		FV		x			(45)			(54)
			FV	x						(48)

* Evidence: Relationship for positive or negative association qualified as statistically significant at the 5% level, based on correlation and regression analysis. Significant differences: tested e.g., via t-tests; ANOVA; No association, no significant difference: not statistically significant, or no association. F, Fruit; FV, Fruit and vegetables combined; V, Vegetables; What: Quantities consumed, amount spent for purchasing FV; % of people consuming F, V or FV; How: Represents the frequency of consumption or purchase; Where: is referring to urban/rural areas; in 70 where is referring to where the consumed FV were obtained from, e.g., from the market or from the wild.

### Individual level

Factors identified at the individual/household level were divided into four sub-levels, including biological, demographic, lifestyle and behavior, and cognition. Altogether, we identified 33 individual-level factors across 45 studies.

#### Biological factors

Biological factors include gender in terms of differences due to biological sex, age, body mass index and pre- to post-menopause comparisons.

##### Gender/biological sex differences

Gender in terms of biological sex differences in fruit, vegetable and combined FV consumption and purchase was investigated in 22 studies (28–30, 32, 33, 35, 39, 40, 42, 46, 48, 50, 52–55, 57, 60, 62, 64, 67, 73). A higher or more frequent intake of fruit, vegetables or combined FV in women than men, was observed across nine studies (30, 33, 39, 40, 46, 52, 54, 64, 67). In four studies the highest intake or purchase of fruit, vegetables or combined FV was observed among men (28, 35, 40, 48). No differences between men and women, neither for fruit, nor for vegetable consumption or purchase was observed in ten studies (29, 32, 42, 50, 53, 55, 57, 60, 62, 73).

##### Age

The relevance of age was examined in 21 studies (28, 29, 31, 32, 35, 40, 42, 43, 46, 50, 52–54, 56, 57, 60, 62, 64, 67, 73, 74). Twelve studies found that the frequency and quantity of fruit, combined FV, and vegetable consumption increased with increasing age (31, 35, 46, 50, 52–54, 56, 60, 64, 67, 73). Two studies found opposing results in which fruit and combined FV consumption decreased with increasing age of consumers (40, 43). One study among adults in Mauritius examined the frequency and also the timing of fruit consumption between age groups and found significant differences between younger and older adults and whether fruit was consumed after lunch or after dinner (74). In seven studies, no association was found between age and fruit; between age and vegetable; or age and combined FV consumption or purchase (28, 29, 32, 42, 50, 57, 62).

##### Other biological factors

The relationship between body mass index and fruit, vegetable and combined FV consumption was examined in three studies (30, 42, 67). One study among urban residents in Ethiopia found positive associations between body mass index and frequency of fruit and vegetable consumption (30), while two studies found no associations (42, 67). One study among women in Mauritius aimed to assess factors affecting food habits between pre-menopausal and post-menopausal women. The results revealed that the consumption of fruit was the highest among pre-menopausal women, whereas raw vegetables were mostly consumed by post-menopausal women (34).

#### Demographic factors

##### Family or household income

The relevance of income was investigated in 15 studies (29, 32, 37, 39, 48, 52, 56, 57, 60, 62, 64, 73, 77, 79, 80). Nine of these studies found that the frequency and quantity of fruit, vegetable and combined FV consumption and combined FV purchase increased with higher family or household income (32, 39, 48, 52, 64, 73, 77, 79, 80). Two studies found positive associations but also opposing results or no associations (64, 79). For example despite a higher intake of fruit, wealthier rural Mozambican women reported a lower consumption of vegetable, while there was no significant variation with income and FV consumption of male or urban respondents (64). Studies discussed that vegetables were components of cheapest meals in rural areas where they grow, while while fruit was more affordable year-round to wealthier families (64). In four studies, household income, or having money was not associated with vegetable purchases or combined FV intake (29, 37, 60, 62).

##### Socio-economic status

The importance of socio-economic status (SES) was assessed in four studies (34, 46, 68, 77). Significant differences between socio-economic status were found among all studies. In one study, significant differences were found for cooked vegetables, but not for vegetable salads (34). Overall results revealed that more people from higher SES consumed fruit and vegetables compared to people from lower SES.

##### Wealth status

The influence of wealth status was investigated in seven studies (31, 40, 43, 44, 67, 77, 80). Six of these studies found that the quantity and frequency of fruit, vegetable or combined FV consumption increased with higher wealth status (31, 43, 44, 67, 77, 80). One study found positive associations but also opposing results (31), showing a decrease in the weekly number of vegetable servings consumed by women in Ghana with increasing wealth status. The relationship was also negative for men, but not significant (31). One study found no association (40).

##### Food insecurity

Food insecurity was assessed in five studies (32, 43, 49, 59, 76). Four studies found that food insecurity was associated with lower or less frequent fruit and vegetable consumption (32, 43, 49, 76). One study found no association between food insecurity and fruit and vegetable purchase among supermarket shoppers from different South African socio-economic communities and discussed that this could be due to the short form of the food security questionnaire used in the study (59). Another study found no association between food insecurity and vegetable consumption, but found that food insecurity was associated with a low amount of fruit consumed during the dry season, while not during the rainy season (76).

##### Education

Twenty studies examined the role of education (29–32, 35, 40, 42, 43, 50, 52–54, 56, 62, 64, 67, 73, 76, 79, 80). Fourteen of these studies found that the frequency and quantity of fruit, vegetable and/or combined FV consumption and purchase increased with higher level of education (30–32, 40, 43, 50, 53, 54, 62, 64, 67, 73, 79, 80). The majority of these studies referred to positive associations between education and fruit consumption for men and women (30, 31, 43, 54, 64, 67, 73, 79). For vegetables, the results were less unambiguous, i.e., more studies showed no associations. Overall, four studies found mixed results, including positive, and opposing results, i.e., higher education was associated with reduced fruit, vegetable, or combined FV consumption (35, 56, 64, 79). Reasons for that contradiction were discussed only in one paper, where vegetable intake was threefold lower in the more educated urban men, while fruit intake was positively associated with education. The authors speculated that families with higher education were more likely to work outside the home, thereby leaving less time for preparing meals which could lead to a greater preference for ready to eat foods including fruit while omitting vegetables (64). Five studies found no associations (29, 42, 52, 67, 76).

##### Occupation/employment

The relevance of occupation or employment status was investigated in 14 studies (28, 31, 32, 39, 42, 43, 50, 53, 54, 56, 57, 73, 79, 80). Nine of these found associations between different types of occupation and fruit, vegetable or combined FV consumption (28, 31, 39, 43, 50, 53, 54, 73, 79). However, no pattern regarding a certain occupation type and its positive or negative relationship with fruit, vegetable or combined FV consumption was observed across the studies. In addition, five studies found no association between employment status and combined FV consumption or purchase behavior (32, 42, 56, 57, 80).

##### Residence

The difference between urban and rural residence in fruit, vegetable, and combined FV consumption was assessed in 11 studies (31, 32, 42, 46, 56, 60, 64, 67, 73, 76, 80). Four studies found that adults living in urban areas consumed less, or less frequently, fruit, vegetables, or combined FV, as compared to adults living in rural areas (31, 64, 67, 73). Four studies found no association between residence and fruit consumption (32, 67, 73, 76), followed by four studies that found no association between residence and combined FV consumption (30, 42, 46, 60), and one study found no association between residence and vegetable consumption (32). Mixed results within studies and within fruit and vegetable groups were observed in two studies relating to biological sex differences, in addition to the difference in urban and rural residence (31, 64).

##### Ethnicity

The influence of ethnicity was assessed in six studies and all of them found associations (31, 32, 46, 54, 67, 73). The results were however inconsistent, depending on which ethnic groups were compared.

#### Lifestyle/behaviors

Within the sub-level “lifestyle/behaviors,” ten factors were identified. Tobacco smoking and drinking habits were the factors investigated by most studies and showed associations with fruit and vegetable consumer behavior, as well as the factors of ease of transportation, vegetarianism, and purchase of sugar-sweetened beverages.

##### Tobacco use/smoking

The factor smoking was assessed in five studies (32, 54, 63, 65, 67). In two of these, smoking compared to non-smoking was associated with a decrease in the amount and frequency of fruit and/or vegetable consumption (63, 65). One study investigated smoking habits in terms of different cigarette types and frequency of tobacco consumption and found negative associations between manufactured cigarette smoking and frequency of fruit and vegetable intake, while also positive association between smokeless tobacco consumption or hand-rolled cigarette smoking and frequency of fruit and vegetable consumption among adults (63). This shows that the negative association between smoking and FV consumption is not the same for all forms of tobacco use. Three studies found no association between smoking and fruit consumption (32, 54, 67); smoking and vegetable consumption (32, 54, 67), or smoking and combined FV intake (54, 67).

##### Alcohol consumption/drinking habits

The relationship between alcohol consumption and the frequency and quantity of fruit, vegetable and combined FV intake was investigated in three studies (32, 54, 67). One study found that drinking was associated with a decrease in combined FV consumption (54). Two studies found no association (32, 67).

##### Travel to purchase groceries

Two studies (combined rural and urban areas) assessed the association between ownership of a vehicle or different modes of travel (e.g., walk, personal vehicle, bus, taxi) to purchase groceries, and FV consumption (60, 73). Results revealed overall positive associations between vehicle ownership or use of a personal vehicle to purchase groceries and fruit, vegetable, and combined FV consumption. Among the discussed reasons was that ownership of a vehicle was considered as a proxy for mobility and ease of transportation, which can enhance the chances of these households accessing cheaper or better-quality FV (73).

##### Access to information technology

The relevance of access to information technology was examined in one study in South Africa (73). Household access to mobile phones, radio, television, and internet was associated with increasing frequency of and higher chances of consuming adequate amounts of fruit and vegetables among adults. The authors argue that access to nutrition information disseminated through various media channels could positively influence nutrition awareness, and point to the promotion of nutritious foods through programs in South Africa, but do not elaborate on specific campaigns, their content, or duration.

##### Other lifestyle factors

The frequency of purchasing sugar-sweetened beverages was associated with a decrease in combined FV consumption (60). The influence of vegetarianism was measured in two studies (32, 56). While one study showed no association between vegetarianism and fruit and vegetable consumption (32), another study showed that vegetarians ate Moringa leaves and pods more often, compared to non-vegetarians (56). Other factors including convenience (45, 68), time (37, 66), physical activity (67), eating out (66), and buying FV daily or weekly (60) and its relationship with fruit and vegetable consumption were examined only by few studies and revealed no associations.

#### Cognition

Nine studies examined the sub-level cognition (37, 45, 48, 50, 56, 60, 66, 68, 76). Five factors, namely, taste preference for vegetables (76), mood (45, 68), higher belief in one's own ability to prepare vegetables (self-efficacy) (68), valuing “health” as food choice motive (68) and attitude toward FV consumption (50) showed positive associations with vegetable and combined FV consumption. The factors knowledge (50, 56, 68) and nutrition education (32, 50), showed mixed results, and personal preferences (37, 45) as well as ethical concern (68) showed no associations with FV consumer behavior.

### Social environment

Thirteen studies explored factors within the social environment which may influence consumer behavior through social interactions, social support or role modeling (12).

#### Family

##### Household size and composition

The role of household size and household composition was investigated in eight studies (29, 32, 40, 50, 53, 73, 76, 79). Household size was most frequently assessed (32, 40, 50, 53, 73, 79). The results revealed that higher household size is associated with less frequent or lower quantity of fruit, vegetables, and combined FV consumption among adults (40, 73, 79). Three studies found no association (29, 32, 53), and three studies found mixed results (40, 50, 73). For example, one study among adults in South Africa found negative associations with fruit consumption, as well as no association between family size and vegetable consumption (73). Another study among adults in rural Tanzania found negative associations between household size and combined FV consumption, as well as a positive association, and no association for specific fruit items (40). And one study in Uganda found a negative association between household size and combined FV consumption in urban, but not in rural areas (50). The composition of the household in terms of the number of adults, the number of females 15 years and older, or the number of children in the household was assessed by three studies (73, 76, 79). One study in South Africa showed overall positive associations between the number of children below 5 years of age and fruit, but not vegetable consumption by adults. In addition, in the same study, the number of females 15 years and older in the households was also positively associated with adults' fruit and vegetable consumption (73). On the contrary, one study in Ghana found a negative association between the number of children in a household and the quantity of fruit consumption among urban dwellers in Ghana, but the results were not further discussed (79). One study among mothers in Malawi found a negative but not significant association between the number of children in a household and the amount of vegetables consumed by mothers (76).

##### Marital status

The factor marital status was examined in nine studies (31, 32, 39, 42, 50, 54, 56, 57, 62). The positive associations between marital status and fruit, vegetable, and combined FV consumption of men and women referred overall to being married or cohabiting vs. not being married (39, 42, 54). One study among adults in Mauritius found an opposing result showing that widowed participants reported higher consumption frequencies of the vegetables “Moringa leaves” and “Moringa pods” compared to those that were single, married or cohabiting (56). Four studies found no associations or no significant differences (31, 32, 57, 62) and one study in Uganda found mixed results, showing a negative association between being married and FV consumption among adults in rural areas, while no association with adults in urban areas (50).

##### Habits and behavior within the family

Factors assessing habits and behavior within the family such as perceived family preferences and eating habits or whether it was the father or mother who purchased food within the family, were sparsely investigated and revealed no association or significant differences in two studies (37, 76).

#### Gender roles and empowerment

The influence of gender roles and empowerment on diets has been investigated in three studies (45, 50, 61). One study among women across five African countries explored the relationship between women's empowerment and the consumption of vegetables and combined FV. Results showed that women's autonomy and input in production decisions were positively associated with the consumption of dark green leafy vegetables, as well as with the consumption of vitamin A-rich FV, while leadership opportunities measured as “women are comfortable speaking in public” was associated only with combined FV consumption (e.g., other fruit and vegetables), but not with dark green leafy vegetables or combined FV (e.g., other vitamin A-rich FV) (61). In one study in urban Ethiopia, “husband's encouragement,” which was described as a social support within the household, was positively associated with women's combined FV consumption (45). One study in Uganda found no association between intra-households decision makings and FV consumption (50).

### Physical environment

Within the physical environment, which includes the different surroundings, where people consume, purchase or acquire food we identified 13 factors divided in the sub-levels availability and access at home, availability at university, neighborhood and retail environment and product property and food safety.

#### Availability and access at home

The importance of the availability of FV in the home for fruit, vegetable, and combined FV consumption was investigated by three studies (37, 45, 73). Two of these studies investigated home-garden/own production for FV consumption once assessed as a binary variable (households engaged in own FV production—yes/no) (73), and once assessed as participants' perception (if participants perceived home-gardens as an enabler for FV consumption) (37). While household engagement in own FV production was associated with more frequent and higher vegetable intake among adults in South Africa (combined urban and rural areas), it showed no association with fruit intake (73). Discussed reasons included that households either produced mainly vegetables or that fruit was sold at the market rather than for own consumption (73). In contrast, home-gardens as a perceived enabler for FV consumption did not enable combined FV consumption among low-income urban residents in Ibadan, Nigeria (37). The same study also examined the influence of storage of FV at home as a perceived enabler for combined FV consumption and found no significant difference between people who consumed and those who did not consume adequate amounts of FV (37). However, women's perception of fruit and vegetable availability in homes was positively associated with adequate combined FV consumption among women in urban Central Amhara Region in Ethiopia (45).

#### Availability at university

The availability of fruit and vegetables at universities and its association with fruit and combined FV purchase among students in Nigeria was explored in two studies (48, 57). While one study showed that availability was positively associated with the amount students spent on fruit per month (57) another study found that availability was negatively associated with combined FV purchases, without further discussing the possible reasons (48).

#### Neighborhood and retail environment

##### Distance to market

The relevance of market access, measured in terms of walking time, km distance of village to market, or as a perceived barrier or enabler for FV consumption was investigated by three studies (37, 43, 76). One study among smallholder women farmers from different agro-ecological zones in rural Western Kenya showed that distance in walking time from home to the closest tarmac road was negatively associated with the weekly fruit consumption of women in the dry season (43). Similarly, one study among women with children less than 5 years in urban and rural Central Regions of Malawi examined market access in terms of minutes to the nearest food market/shop and also found a negative, but not significant association with the amount of fruit consumed by women during the dry season (76). Among low-income urban residents in Ibadan, Nigeria, the market access assessed was not detected as a significant determinant for adequate FV intake (37).

##### Availability of FV in the neighborhood

Availability of FV in the neighborhood as a perceived enabler or barrier to FV consumption was explored in one study among low-income residents in Ibadan, Nigeria, but revealed no significant difference between adults who consumed adequate amounts of FV daily, and those who did not (37).

##### Socio-economic areas

The interplay between socio-economic areas and the food purchasing behavior of urban supermarket shoppers was investigated by one study, reported in two publications in South Africa (58, 59). Results revealed that urban supermarket shoppers living in low socio-economic neighborhoods purchased fruit and vegetables less frequently than shoppers from high and middle socio-economic areas (58). Moreover, shoppers from high socio-economic areas spent a significantly higher proportion of their expenditure on fruit compared to shoppers from low and middle-income socio-economic areas (59).

##### Supermarkets

The consequences of modernizing retail environments investigated as the effect of supermarkets on consumers' diets were assessed by one study in three towns in Kenya. The results showed that shopping in supermarkets contributed to a significant decrease in energy consumption from FV among adults (38).

##### Price

The relevance of price was investigated in three studies among urban consumers in Nigeria and Ethiopia (37, 45, 68). Price was found to be the only determinant of combined daily FV consumption among low-income residents in Ibadan, Nigeria (37). Another study among urban women in Nigeria found that price was considered an important food choice motive, overall for women from lower socio-economic status, however, no association was found with vegetable intake (68). Similarly, concerns about food prices were mentioned as a key driver of food choice among women in urban central Amhara region, Ethiopia, but was not associated with the combined FV intake of the women (45).

#### Product property and food safety

The importance of product properties as factors affecting FV consumption and vegetable purchase among adults was assessed in two studies in Ibadan, Nigeria (29, 37). One study (29) examined whether the preferred size or the preferred type/variety of fresh tomato was associated with the weekly amount spent on fresh tomatoes. The results showed that the size of the tomato (medium compared to others) was positively associated with the weekly amount spent on fresh tomatoes, while other variables including the type/variety of fresh tomatoes showed no association (29). Poor product quality as a perceived barrier showed no significant difference between low-income residents in Ibadan, Nigeria, who consumed five portions of FV daily, and those who did not (37). The role of consumers' confidence in food safety actions for vegetables sold in open markets and how it influences the vegetable handling of adults at home was investigated by one study in urban Ghana (47). Results revealed that a higher confidence in food safety actions related to cleanliness and contact exposure, increased the probability of delayed consumption of vegetables and treatment of vegetables at home (47).

### Macro-level environment

Nineteen studies investigated the role of the macro environment, which has a more distant and indirect, but powerful role in influencing consumer behavior.

#### Season

Seasonal differences in fruit, vegetable, and combined FV consumption or acquisition were investigated in eight studies (36, 40, 41, 43, 69, 70, 72, 76). Six out of eight studies found significant differences in the quantity and frequency overall of vegetable consumption (36, 41, 43, 69, 70, 72, 76), followed by fruit (41, 43, 69, 72, 76) and combined FV (41, 43, 70) consumption among adults between seasons. Besides the quantity and frequency of fruit and vegetable consumption, one study assessed whether seasonality influenced “where” fruit and vegetables were obtained for consumption, differentiating between “cultivated,” “from the wild” or “from the market” (70). Results showed that in the rainy season, where fruit and vegetables were overall less frequently consumed, the acquisition of fruit and vegetables “from the wild” as well as “from cultivation” was crucial for the supply compared to “from the market.” The majority of the studies analyzed seasonal variations in rural areas (36, 40, 43, 69, 70, 72) and one study determined the influence of season in rural and urban settings (41). Seasonal differences were mostly expressed as a comparison between two seasons, e.g., rainy vs. dry season, lean vs. post-harvest, or beginning of cereal shortage season vs. to end of cereal shortage season (36, 40, 43, 69, 70, 72, 76). One study analyzed the difference between three agricultural seasons, harvest, post-harvest, and lean season (41).

#### Natural landscape

Within the sub-level natural landscape, the role of ecological zones as well as forests in terms of forest cover and proximity to forests was assessed among three studies (31, 40, 75). The association between ecological zones and fruit and vegetable consumption was examined by one study in Ghana and revealed that adults living in Forest zones consumed more weekly fruit and vegetable servings than those from the Coastal and Savannah zones (31). One study in rural Tanzania assessed whether deforestation over a five-year period affected people's dietary quality including per capita consumption of fruit, vegetables, and combined FV (40). The authors used a modeling approach based on secondary data and showed that forest cover was positively associated with per capita consumption of the food group “fruit and vegetables.” The authors argue that deforestation most likely reduced the local supply for gathering and consuming wild fruit and vegetables in the selected study area. In addition, the authors analyzed individual fruit and vegetable categories responsible for this relationship and showed positive associations between forest cover and the vegetable group “spinach, cabbage, and other green vegetables,” as well as the fruit group “mango, avocado, and other fruit.” Forest cover was, however, not associated with any other fruit or vegetable category (40). In one cross-sectional study in Southwest Cameroon, women of reproductive age from forest-based villages were more likely to consume vitamin A-rich fruit and vegetables than women from non-forest-based villages, while no significant differences were observed for other fruit and dark green leafy vegetables (75).

#### Urbanization

Urbanization in terms of strata of urbanization, and rural-to-urban migration and urbanicity level in rural areas, was investigated in three studies (51, 71, 78). One study among men and women living in the North West Province of South Africa found significant differences among the strata of urbanization (rural, farm, informal settlement, middle class, urban, upper class urban) and fruit and vegetable consumption of adults (51). Another study in Tanzania investigated changes in diet among adults migrating from rural to urban Tanzania over 12 months and found that rural-to-urban migration led to a significant increase in the weekly number of combined FV portions consumed by women, but not by men (78). On the contrary, one study in Uganda that examined the distribution of urban characteristics across rural communities found that higher urbanicity was associated with lower combined FV consumption among adults (71).

#### Cultural and societal norms

The role of religion was investigated in four studies (31, 45, 48, 54). Three of these studies analyzed religion and its association with fruit, vegetable, or combined FV consumption, and one study looked at combined FV purchase. Two studies found associations between religion and fruit, vegetable, and combined FV consumption, one among adults in Ghana (31) and one among urban residents in Central Amhara, Ethiopia (45). One argument in the study in urban Ethiopia on why religious practices are associated with FV consumption was that fruit and vegetables are fasting foods and consumed in the fasting time especially by people who belong to the Orthodox religion, which was most of the women in the study area (45).

Two studies found no association between religion and fruit and vegetable consumption (48, 54).

## Discussion

To the best of our knowledge, this review is the first and most current comprehensive synthesis of factors, identified across four levels of a socio-ecological framework that has been contextualized to the fruit and vegetable consumption and purchase behavior of adults in sub-Saharan Africa. Most evidence in our review was found for demographic factors at the individual/household level. Due to the focus on individual/household level factors, we identified research gaps in the other levels of influence (social, physical, macro), which is consistent with previous reviews in urban Africa (11, 20, 22, 81) and LMICs (82, 83). Nevertheless, we found important evidence for several key variables in the social, physical and macro-level environment, which emphasizes the need for holistic, systemic approaches to promote FV consumption.

### Individual, social, physical and macro level—Where is the evidence?

Most consistent evidence within the individual/household level exists for demographic factors including household or family income, socio-economic status and wealth status which were mostly all positively associated with adults' fruit and vegetable consumption and purchase. These variables are often used as proxy for affordability and demonstrate that equity issues are key among individuals and households in accessing fruit and vegetable. The results are not surprising as affordability, defined as the cost of diets or price relative to income, is known as critical barrier to the consumption of fruit and vegetables, as these foods are among the most expensive components in diets in LMIC in particular (84–87). The consumption of fruit and vegetables is particularly unaffordable for many people from low-income countries including in Africa (86). While it is indisputable that fruit and vegetables must first be made available and affordable for everyone, additional factors including individual preferences, taste, convenience, as well as time are regarded as important drivers of choice among affordable items (18, 88), but these aspects have only been sparsely investigated in the included studies.

Within the social environment, the most consistent evidence exists for household size and marital status, while family habits or interaction within the family or community were rarely assessed. Evidence for household size showed that increasing size was related to lower or less frequent fruit and vegetable consumption. This implies that larger households require more resources to provide for the needs of all household members than smaller households, and are therefore less likely to consume adequate amounts of fruit and vegetables (73). With regards to marital status, some evidence exists that being married or cohabiting is associated with higher and more frequent fruit and vegetable consumption. Authors argued that marriage involve social interactions including regular meals, as well as possible control over the health behavior of the spouse (42). While evidence exists in the wider literature that gender equality and women's empowerment can lead to better food security, nutrition and sustainable food systems (89), only three studies included in our review examined these issues. Evidence from two studies showed positive associations between women's autonomy and input in production decisions, leadership opportunities and husbands encouragement explained as “social support” within the household and women's FV consumption (45, 61). A possible explanation for the lack of research, might be that gender aspects are assessed in relation to other measures, such as dietary diversity (90) or household nutrition (91) and not in relation to specific food items at individual level. Furthermore, intra-household relations and empowerment are difficult to assess with quantitative measures only (92, 93).

Similarly, as for the social environment, evidence in the physical environment was only scattered around a few variables. A potential explanation is that research on food environments has rarely been studied in LMICs, especially in Africa and is only yet emerging (81, 94). Nevertheless, we found some evidence to support arguments that (i) the rapidly changing physical environment in urban areas leads to shifts in the availability and types of food consumed (81, 95, 96) and (ii) that supermarkets do not necessarily provide access to healthy and affordable food (95). This was confirmed by a panel data study in three Kenyan cities, which showed that that shopping in supermarkets contributed to lower consumption of FV, but higher consumption of processed and highly processed foods (38). Authors argued that unprocessed foods like FV are hardly sold in small-town supermarkets in Kenya, compared to processed foods, because they are available from local wet markets (38). Another study discussed issues of FV quality in supermarkets and the general higher prices of FV compared to staples and snacks as a possible reason why urban supermarket shoppers in low socio-economic neighborhoods in urban South Africa purchased fruit and vegetables less frequently than shoppers from high and middle socio-economic areas (58, 59). While food safety concerns are growing barriers to fruit and vegetable consumption in urban LMIC settings (83), we found only a few studies on these aspects in our review.

At the macro-level environment, seasonality was the most frequently studied factor and results were consistent across studies showing significant differences in FV consumption between seasons. Among the main arguments within the studies was that seasonality is a crucial element of food availability, particularly in rural areas, where smallholder farm households depend on rainfed agricultural production. Moreover, seasonality leads to price fluctuations, particularly in Africa, affecting overall perishable food like fruit and vegetables (97). Additional related factors and evidence found at macro-level include the importance of the natural landscape including forests for fruit and vegetable consumption by overcoming seasonal gaps in subsistence settings, but also by providing fresh fruit and vegetables at local markets (31, 40, 75). We found no studies on other factors that are known to influence dietary behavior at the macro level including advertising and marketing, agricultural policies, subsidies or distribution systems.

### Research recommendations

Our analysis reveals some issues regarding research methodology and metrics applied for exposure and outcome variables and allows us to provide some recommendations for future research. See also Box 1 Key messages for future research.

Box 1 **Key messages for future research.**

**Study population**

> Need for more gender- differentiated studies including both men and women in different social, economic and geographic contexts

**Exposure/factors**

Need for more research on:

> preferences, perceptions attitudes as well as on time and convenience aspects at the individual level

> habits and behavior within the family, social identity, social networks, gender equality and women's empowerment at the social environment level

> food safety concern and interactions within the diverse physical food environments

> advertising and marketing of FV, agricultural policies, subsidies or distribution systems of FV

**Outcome**

> Need for more research beyond dietary intake (frequency and quantity of FV), assessing consumer behavior in terms of how, where, when FV are consumed, purchased, acquired or gathered

> Need for more diverse classification of fruit and vegetables, beyond the level of food groups

> Need for more tools and standardized indicators for exposure and outcome variables, and different types of research methodologies and approaches, including qualitative and participatory research methods (see Research Recommendations)

#### Need for new tools and standardized indicators

We observed an absence of metrics and indicators to assess exposure variables across the different levels of influence. For example, “distance to markets” included measures such as “walking time” or “kilometer distance,” as well as asking consumers about their “perception of market access.” This makes comparisons across studies difficult. The lack of standardized indicators and tools is consistent with findings from previous reviews on food environment research in LMICs (19, 94). Downs et al. (19) provide a toolbox of objective and subjective tools to overcome this gap, but highlight that new tools and methods are needed to assess the diverse food environment landscapes in LMICs. With regards to the outcome variables, we found few studies that assessed consumer behavior other than dietary intake. Similarly, as for the exposure variables, reasons for this absence include a lack of validated metrics and indicators to assess consumer behavior, as pointed out in the literature (98).

#### Need for different types of research methodologies

The focus on “objective” observable facts clearly highlights how limited the positivist paradigm is in studying influences on consumer behavior, as reflected in the limited research we have identified on the social, physical, and macro level environment. Moreover, following a conventional hierarchy of evidence only reflects the dominant scientific view, while other knowledges including indigenous knowledge systems, which are key particularly for understanding context specific issues, are left out (99). Several exposure variables can be measured objectively and require standardized indicators. However, other aspects of influence, which are influenced by contextual factors such as habits and behavior within the family, social identity, social networks, interactions within the food environments, or individual perceptions require different types of research methodologies (19, 82, 92), which were not included in this review. Hence as suggested in recent reviews and the literature, to better explore the social and physical environments, different approaches are recommended, that bring the perspective of the consumers to the forefront, such as photovoice or transect walks and other participatory methods (15, 19, 20, 100, 101).

#### Need to address underlying and structural issues

In order to achieve healthy, sustainable and just transformations of food systems, underlying political and structural issues of food environments, of inequity and power imbalances should not be neglected (102, 103). Global food trade and transnational food corporations determine what food is available, affordable or advertised in local food environments of LMIC, which should be taken into account when assessing FV intakes (102). Crucial factors related to increasing local production diversity, such as farmers' access to seeds and exchange of planting materials or land tenure issues (104) were not captured in the reviewed literature. Reasons for might include the focus of this review on observational, overall cross-sectional studies, but also the restriction of outcome variables to consumption and purchase behavior. We could have found studies on these topics, by either adding additional outcome variables such as acquisition, gathering or production of FV or by including qualitative studies. The need to address political economy drivers to transform food systems is increasingly emphasized in the wider literature (102, 103). Scholars from feminist theories, food sovereignty and right-to-food activists emphasize the importance of knowledge co-production with actors outside of academia, giving a voice to marginalized groups, to address issues of inequity and power imbalances (105).

#### Need for more diverse classification of fruit and vegetables

Fruit and vegetables were mainly assessed at the food group level and information on single food items at the species level or below species level, i.e., at cultivar level or on indigenous fruit or vegetables species was mostly lacking. This is unfortunate as it undermines the importance of agricultural biodiversity in local food systems, which plays a central role in supporting and strengthening food, nutrition, health and livelihood security, overall in rural subsistence settings (106). The limitation has also been highlighted in recent reviews on vegetables for healthy diets (107) and in a review on biodiversity in food consumption studies (108). Harris et al. (107) argue that a higher nuance in classifying vegetables related to dietary outcomes is needed to assess the diversity within food groups. We support this argument which should also be extended for fruit, while also considering local species including indigenous and orphan crops.

#### Policy recommendations

Despite the paucity of evidence due to a lack of research across the different levels of influence, the review identified some policy recommendations. To address issues of economic access to fruit and vegetable consumption, interventions aimed at reaching lower socio-economic groups, such as social protection programs improving access to credit or voucher systems have been suggested by studies in this review (37) and in the wider literature (84). Moreover, making FV more affordable was further discussed as a regulatory strategy in articles included in this review (68) and in other literature (11, 84, 87, 109). Recommended actions to lower the prices discussed in the wider literature encompass subsidies on fruit and vegetable production, as well as improving local production, marketing, trade, and storage (11, 84, 87, 109). Incentivizing the sale of healthier foods, such as fruit and vegetables in retail markets has also been suggested in included studies (38). However, as formal retail outlets are often competitive with informal food economies, context-specific solutions are required (95, 96). For example, an approach discussed in the literature is to support traditional markets, including wet markets and farmers' markets that sell fresh products around supermarkets, which can support the livelihoods of small informal vendors that might be replaced by large retail outlets (83, 95, 96). Supporting the sale of FV through small vendors could also improve access to FV since supermarkets are often out of reach especially for lower socio-economic groups.

To ensure the year-round harvest of FV overall in subsistence settings, location-specific production calendars with a focus on trees and shrubs adapted to agro-ecological conditions have been suggested as solutions by studies in this review (43) and in the wider literature (110). Other strategies mentioned included focusing on improved methods of food storage and processing techniques for FV to maintain dietary diversity (41), and to improve the utilization of FV in value chain developments (43). In addition, gathering fruit and vegetables from the wild, from near forests was mentioned as coping strategy to overcome seasonal unavailability of FV among studies within the review (70, 75). Local production of fruit and vegetables has the potential for direct consumption in subsistence settings. In addition, production at the local landscape can ensure access to the nutritious, but perishable FV in local markets, especially in areas where infrastructure is not well developed (overall rural), thus avoiding seasonal price fluctuations (111). Moreover, in order to sustainably transform our food systems, scientists have emphasized the importance to recognize, protect and support forests and agroforestry landscapes in the discourse around food and nutrition security. These systems are important suppliers particularly of FV, and provide ecosystem services essential for producing other food (111–114).

### Strengths and limitations of the review approach

This review has several strengths and limitations. One strength is that we followed a systematic review methodology with a comprehensive search in the electronic databases Scopus, PubMed, PsycINFO, African Index Medicus, and Google Scholar. While previous reviews in Africa assessed factors on general dietary behavior, limited to urban areas (11, 20, 22), we focused on the specific food categories fruit and vegetables and included both urban and rural settings. In addition to exposure and outcome associations, we included descriptive studies, if significance tests were presented. This allowed us to include a wide range of potential factors, such as the most studied factor at the macro level (seasonality) which was mainly assessed *via* descriptive statistics, lacking the assessment of potential confounders. This review is a synthesis of observational studies, with overall cross-sectional study design, as this type of studies was predominant in an initial scoping search. Nevertheless, cross-sectional studies provide only a snapshot of the present moment and do not allow conclusive statements on causality between exposure and outcome. We performed a critical appraisal for each study to identify potential bias, but did not rate the quality of evidence. This is a limitation of our review, because it is recommended to not only base evidence evaluations on statistical significance, but to consider the strengths of the association and other aspects that could lead to imprecision or inconsistency (115). Another limitation is that only English studies were included, which restricted the inclusion of studies in French or Portuguese speaking African regions, which is reflected in the geographic distribution. We found most studies were located in East and Southern Africa, but few in West and Central Africa. The restriction to individual level outcome measures excluded many purchase outcomes, which might have covered more aspects in the physical environment.

## Conclusion

This review fills a knowledge gap to better understand the various factors that enable or constrain fruit and vegetable consumption and purchase among adults in sub-Saharan Africa. Most consistent evidence was found at the individual/household level for demographic factors including household or family income and socio-economic status. While fewer studies assessed other levels of influence, we found important evidence for several factors at the social, physical, and macro levels. These include the importance of women's empowerment, the influence of neighborhood and food retail environment including distance to market and price, and the importance of natural landscapes, including forest areas, on consumption of FV. This underscores the need for context-specific approaches at multiple levels to promote FV consumption. The lack of evidence, particularly on aspects such as social interaction within the family, community, or food environment, as well as consumer behavior beyond dietary intake, was identified as a limitation. It highlights the need to develop and improve indicators for both exposure and outcome variables, but also the need to diversify research approaches to reflect not only the dominant scientific view but also to include other knowledge, including indigenous knowledge systems, that are, particularly critical to understanding context-specific issues.

## Data availability statement

The original contributions presented in the study are included in the article/Supplementary material, further inquiries can be directed to the corresponding author.

## Author contributions

BS with contributions from UT, IB, IS, SM, MW, LH, and AK worked on the conceptualization of the review. UT and IS provided guidance on the methodology of the review process. BS conducted the literature search, screened all articles, extracted data, conducted data analysis, wrote the original draft, and integrated feedback from co-authors in the final version. UT, IS, AK, LH, and SM assisted BS in the double screening of articles and checks in data extraction and risk of bias assessment. SL, UT, and IS provided substantial feedback in the draft and final version. SL, UT, MW, IS, SM, and PR contributed review and editing. All authors read and approved the final version of the manuscript.
