# Supplementary material for: Factors affecting fruit and vegetable consumption and purchase behavior of adults in sub-Saharan Africa: A rapid review
Source: Front Nutr. 2023 Apr 11;10:1113013. doi: 10.3389/fnut.2023.1113013 (PMC10126510; doi:10.3389/fnut.2023.1113013)
Supplement: Supplementary file 1 [file Data_Sheet_1.PDF]

Supplementary Material S1

Search: 2000 to April 2022

Search Example in SCOPUS

	No	Scopus Syntax
Outcomes	#1	(TITLE-ABS-KEY (fruit*) OR TITLE-ABS-KEY (vegetable*)) AND (TITLE-ABS-KEY (diet*) OR TITLE-ABS-KEY (consum*) OR TITLE-ABS-KEY (eat*) OR TITLE-ABS-KEY (intake*) OR TITLE-ABS-KEY (buy*) OR TITLE-ABS-KEY (purcha*) OR TITLE-ABS-KEY (acqui*) OR TITLE-ABS-KEY (“food expenditure”) OR TITLE-ABS-KEY (“food practice*”) OR TITLE-ABS-KEY (“dietary practice*”) OR TITLE-ABS-KEY (“eating practice*”) OR TITLE-ABS-KEY (*behaviour*))
Context (sub-Saharan Africa)	#2	TITLE-ABS-KEY (Angola OR Benin OR Botswana OR “Burkina Faso” OR Burundi OR Cameroon OR Cameron OR “Cabo Verde” OR “Cape verde” OR “Central African Republic” OR Chad OR Comoros OR Comoro OR Comores OR “Côte d’Ivoire” OR “Ivory Coast” OR “Democratic Republic of Congo” OR “Republic of Congo” OR Congo OR Guinea OR “Guinea-Bissau” OR “Equatorial Guinea” OR Eritrea OR Eswatini OR Swaziland OR Ethiopia OR Gabon OR Ghana OR Gambia OR Kenya OR Lesotho OR Liberia OR Madagascar OR Malawi OR Mali OR Mauritania OR Mauritius OR Mozambique OR Namibia OR Niger OR Nigeria OR Rwanda OR Ruanda OR “Sao Tome and Principe” OR Senegal OR Seychelles OR “Sierra Leone” OR Somalia OR “South Africa” OR “South Sudan” OR Sudan OR Tanzania OR Togo OR Uganda OR Zambia OR Zimbabwe)
	#3	TITLE-ABS-KEY (Africa)
Context	#4	#2 OR #3
	#5	#1 AND #4
Language	#6	AND (LIMIT-TO (LANGUAGE , “English”)
Year		2000 to 2022

Medline/PubMED

	No	Pubmed Syntax
Outcomes	#1	Search (“Fruit"[MeSH Terms] OR "Vegetables"[MeSH Terms] OR fruit* OR vegetable*) AND ("Diet" [Mesh] OR diet*[tiab] OR intake [tiab] OR consum* [tiab] OR eat [tiab] OR eating [tiab] OR purchas* [tiab] OR buy [tiab] OR acqui*[tiab] OR food expenditure [tiab] OR food practice [tiab] OR dietary practice [tiab] OR eating practice [tiab] OR behaviour*[tiab] OR "Consumer Behavior"[Mesh])
Context (sub-Saharan Africa)	#2	Search (Angola OR Benin OR Botswana OR "Burkina Faso" OR Burundi OR Cameroon OR Cameron OR "Cabo Verde*" OR "Cape verde" OR "Central African Republic" OR Chad OR Comoros OR Comoro OR Comores OR "Côte d'Ivoire" OR "Ivory Coast" OR "Democratic Republic of Congo" OR "Republic of Congo" OR Congo OR Guinea OR "Guinea-Bissau" OR "Equatorial Guinea" OR Eritrea OR Eswatini OR Swaziland OR Ethiopia OR Gabon OR Ghana OR Gambia OR Guinea OR "Guinea-Bissau" OR Kenya OR Lesotho OR Liberia OR Madagascar OR Malawi OR Mali OR Mauritania OR Mauritius OR Mozambique OR Namibia OR Niger OR Nigeria OR Rwanda OR Ruanda OR "Sao Tome and Principe" OR Senegal OR Seychelles OR "Sierra Leone" OR Somalia OR "South Africa" OR "South Sudan" OR Sudan OR Tanzania OR Togo OR Uganda OR Zambia OR Zimbabwe)
	#3	Search (Africa [tiab])
Context	#4	#2 OR #3
	#5	#1 AND #4
Language	#6	AND (LIMIT-TO (LANGUAGE , "English")
Year	#7	2000 – 2022
Limit to Humans	#8	Search "Animals"[Mesh] NOT "Humans"[Mesh]
Final	#9	Search (#7 NOT #8)

Supplementary Material S1 Quality assessment

1. Risk of bias –Cross Sectionals studies

The risk of bias was assessed alongside the data extraction process using the Appraisal tool for Cross-Sectional Studies (AXIS) (26).

1. Were the aims/objectives of the study clear?
2. Was the study design appropriate for the stated aim(s)
3.Was the sample size justified?
4.Was the target/reference population clearly defined?
5.Was the sample frame taken from an appropriate population base so that it closely represented the target/reference population under investigation?
6.Was the selection process likely to select subjects/participants that were representative of the target/reference population under investigation?
7.Were measures undertaken to address and categorize non-responders?
8.Were the risk factor and outcome variables measured appropriate to the aims of the study?
9.Were the risk factor and outcome variables measured correctly using instruments/measurements that had been trailed, piloted or published previously?
10. Is it clear what was used to determined statistical significance and/or precision estimates?
11. Were the methods (including statistical methods) sufficiently described to enable them to be repeated?
12. Were the basic data adequately described?
13. Does the response rate raise concerns about non-response bias?
14. Were the results internally consistent?
15. Were the results for the analyses described in the methods, presented?
16. Were the authors' discussions and conclusions justified by the results?
17. Were the limitations of the study discussed?
18. There were no funding sources or conflicts of interest that may affect the authors' interpretation of the results
19. Was ethical approval or consent of participants attained?

✓	If the study clearly met the criteria
x	If the study clearly did not achieve the criteria
?	If the study did not provide enough information to determine a yes/no scoring clearly

Risk of bias cross-sectional studies (primary studies)

Authors	1	2	3	4	5	6	7	8	9	10	11	12	13	14	15	16	17	18	19	Overall risk of bias
Adeoye et al., 2016	✓	✓	✓	✓	✓	?	x	?	?	x	x	x	?	✓	✓	x	x	?	?	High
Adenegan et al., 2018	✓	✓	?	✓	?	?	x	?	?	x	?	x	?	?	?	x	x	?	?	High
Amare et al., 2012	✓	✓	✓	✓	✓	✓	✓	✓	✓	✓	?	✓	✓	✓	✓	✓	✓	✓	✓	Low
Badurally et al., 2012	✓	✓	x	✓	?	?	x	?	?	✓	✓	x	?	x	x	?	x	?	✓	High
Banwat et al., 2012	✓	✓	✓	✓	✓	?	x	?	?	✓	✓	x	?	?	?	?	x	?	✓	High
Bhurosy, T., Jeewon, R.	✓	✓	✓	✓	?	✓	x	✓	✓	✓	✓	✓	?	✓	✓	✓	x	✓	✓	Moderate
Bloomfield et al., 2013	✓	✓	?	✓	✓	✓	✓	?	✓	✓	✓	✓	✓	✓	✓	✓	✓	✓	✓	Low
De Filippo et al., 2021	✓	✓	x	✓	?	✓	x	✓	✓	✓	✓	✓	?	✓	✓	✓	✓	✓	✓	Low
Gelibo et al., 2017	✓	✓	✓	✓	✓	✓	✓	?	✓	✓	✓	✓	✓	✓	✓	x	x	✓	?	Moderate
Kibr et al., 2021	✓	✓	✓	✓	x	✓	x	✓	✓	✓	✓	✓	?	✓	✓	?	✓	✓	✓	Moderate
Labadarios et al., 2011	✓	✓	?	✓	✓	✓	?	✓	✓	✓	x	✓	?	✓	✓	?	x	✓	✓	Moderate
Lagerkvist et al., 2018	✓	✓	x	✓	?	?	x	✓	✓	✓	✓	✓	?	✓	✓	✓	✓	✓	?	Moderate
Layade et al., 2014	✓	✓	x	✓	✓	?	x	x	?	x	x	x	?	x	✓	x	x	✓	?	High
Leyna et al., 2010	✓	✓	✓	✓	x	?	x	✓	✓	✓	✓	?	?	?	✓	✓	✓	✓	✓	Moderate
Lomira et al., 2021	✓	✓	✓	?	✓	✓	x	✓	✓	✓	✓	?	✓	✓	✓	✓	✓	✓	✓	Moderate
MacIntyre et al., 2002	✓	✓	?	✓	✓	?	x	✓	✓	✓	✓	✓	?	✓	✓	✓	x	✓	✓	Low
Modibedi et al., 2021	✓	✓	?	✓	✓	?	x	✓	?	✓	✓	✓	?	✓	✓	✓	x	?	?	Moderate
Musaiger et al., 2016	✓	✓	?	✓	x	?	✓	?	?	✓	✓	?	?	✓	✓	✓	x	✓	✓	Moderate
Neergheen-Bhujun et al., 2020	✓	✓	?	✓	?	✓	x	✓	✓	✓	✓	?	?	✓	✓	?	x	?	✓	Moderate
Obayelu et al., 2019	✓	✓	x	✓	?	?	x	x	?	✓	x	x	?	?	✓	?	x	?	?	high
Odunitan-Wayas et al., 2018	✓	✓	x	✓	x	✓	✓	?	✓	✓	✓	✓	?	x	✓	✓	✓	✓	✓	Moderate
Odunitan-Wayas et al., 2020	✓	✓	x	✓	x	✓	✓	?	✓	✓	✓	✓	?	✓	✓	✓	✓	✓	✓	Moderate
Okop et al., 2019	✓	✓	✓	✓	x	?	?	?	✓	✓	✓	✓	?	?	✓	x	✓	✓	✓	Moderate
Oyedele et al. 2018	✓	✓	?	✓	?	?	x	x	?	✓	x	x	?	✓	✓	x	x	✓	?	High
Padrão et al., 2011	✓	✓	x	✓	✓	✓	✓	✓	?	✓	✓	✓	✓	✓	✓	✓	✓	✓	✓	Moderate
Padrão et al., 2012	✓	✓	✓	✓	✓	✓	✓	?	✓	✓	✓	✓	✓	✓	✓	✓	✓	✓	✓	Low
Peltzer et al., 2004	✓	✓	x	✓	x	✓	x	?	✓	x	x	✓	?	✓	✓	x	x	✓	✓	Moderate
Raaijmakers et al., 2018	✓	✓	x	✓	?	?	x	✓	✓	x	x	✓	?	x	✓	✓	✓	✓	?	Moderate
Sinyolo et al., 2020	✓	✓	?	✓	✓	✓	x	?	?	✓	?	?	?	✓	✓	x	✓	✓	?	Moderate
Subratty and Jowaheer, 2001	✓	✓	?	✓	?	?	x	?	x	x	x	x	?	✓	x	x	x	?	?	High
Tata et al., 2019	✓	✓	✓	✓	?	✓	x	✓	✓	✓	✓	✓	?	✓	x	✓	✓	✓	✓	Moderate

Torheim et al., 2004	✓	✓	✓	✓	?	✓	x	✓	✓	✓	✓	✓	?	✓	✓	✓	x	?	✓	Moderate
Wang et al., 2016	✓	✓	x	x	x	?	x	?	x	x	x	?	?	x	✓	x	x	?	?	High

Risk of bias cross-sectional studies (secondary studies)

Authors	1	2	3	4	5	6	7	8	9	10	11	12	13	14	15	16	17	18	19	Risk of bias
Amo-Adjei et al., 2015	✓	✓	NA	✓	✓	✓	✓	?	✓	✓	x	✓	✓	✓	✓	✓	✓	?	NA	Moderate
Kabwama et al., 2019	✓	✓	NA	✓	✓	?	✓	✓	✓	✓	✓	✓	✓	✓	✓	✓	✓	✓	NA	Low
Keetile et al., 2019	✓	✓	NA	✓	?	?	✓	✓	?	✓	✓	?	✓	✓	✓	✓	✓	✓	NA	Moderate
Mayén et al., 2016	✓	✓	NA	✓	✓	?	✓	✓	?	✓	✓	?	?	✓	✓	✓	✓	?	NA	Low
Msambichaka et al., 2018	✓	✓	NA	✓	?	?	?	✓	✓	✓	✓	?	?	✓	✓	✓	✓	✓	NA	Moderate
Onah et al., 2021	✓	✓	NA	✓	?	?	?	✓	?	✓	✓	?	?	✓	✓	✓	✓	✓	NA	Moderate
Peltzer and Pengpid, 2020	✓	✓	NA	✓	✓	?	✓	✓	✓	✓	✓	✓	✓	✓	✓	✓	✓	✓	NA	Low
Pengpid and Peltzer, 2018	✓	✓	NA	✓	?	?	✓	?	?	✓	x	?	✓	✓	NO	✓	✓	✓	NA	Moderate
Riha et al., 2014	✓	✓	NA	✓	✓	?	✓	✓	✓	✓	✓	✓	?	✓	✓	✓	✓	✓	NA	Low
Yaya et al., 2018	✓	✓	NA	✓	✓	?	✓	x	?	✓	x	✓	?	✓	NO	NO	✓	✓	NA	Moderate

2. Risk of bias – Longitudinal studies

For longitudinal studies, we adapted the Appraisal tool for Cross-Sectional Studies (AXIS) (26) was used with question from the Quality Assessment Tool for Quantitative Studies" developed by the Effective Public Health Practice Project (EPHPP) (27).

1. Were the aims/objectives of the study clear?<br>2. Was the study design appropriate for the stated aim(s)<br>3.Was the sample size justified?<br>4.Was the target/reference population clearly defined?<br>5.Was the sample frame taken from an appropriate population base so that it closely represented the target/reference population under investigation?<br>6.Was the selection process likely to select subjects/participants that were representative of the target/reference population under investigation?<br>7.Were the risk factor and outcome variables measured appropriate to the aims of the study?<br>8.Were the risk factor and outcome variables measured correctly using instruments/measurements that had been trialled, piloted or published previously?<br>9. Is it clear what was used to determined statistical significance and/or precision estimates?<br>10. Were the methods (including statistical methods) sufficiently described to enable them to be repeated?<br>11. Were the basic data adequately described?<br>12. Were the results internally consistent?<br>13. Were the results for the analyses described in the methods, presented?<br>14. Were the authors' discussions and conclusions justified by the results?<br>15. Were the limitations of the study discussed?<br>16. There were no funding sources or conflicts of interest that may affect the authors' interpretation of the results<br>17. Was ethical approval or consent of participants attained?<br>18. Were withdrawals and drop-outs reported in terms of numbers and/or reasons per group?<br>19. Indicate the percentage of participants completing the study. Answer options: 1) 80 -100%; 2) 60 -79%; 3) less than 60%; 4) Can't tell; 5) Not Applicable

Authors	1	2	3	4	5	6	7	8	9	10	11	12	13	14	15	16	17	18	19	Overall risk of bias
Bosha et al., 2019	✓	✓	✓	✓	?	✓	✓	✓	✓	✓	✓	✓	✓	?	✓	✓	✓	?	?	Moderate
Jordan et al., 2022	✓	✓	✓	✓	?	✓	✓	✓	✓	✓	✓	✓	✓	✓	✓	✓	✓	✓	80-100 %	Low
Keding et al., 2017	✓	✓	x	✓	✓	✓	✓	✓	✓	✓	✓	✓	✓	✓	x	✓	✓	✓	60- 79%	Low
Ravaoarisoa et al., 2019	✓	✓	✓	✓	✓	✓	✓	✓	✓	✓	✓	✓	✓	?	x	✓	✓	✓	80-100 %	Low
Reyes-García et al., 2019	✓	✓	x	✓	?	✓	✓	✓	✓	✓	✓	✓	✓	✓	x	✓	✓	x	?	Low
Savy et al., 2006	✓	✓	x	✓	?	✓	✓	✓	✓	✓	✓	✓	?	✓	?	?	✓	✓	80-100 %	Low
Thakwalakwa et al., 2019	✓	✓	x	✓	✓	?	✓	✓	✓	✓	✓	✓	✓	✓	x	✓	✓	✓	80-100 %	Low
Unwin et al., 2010	✓	✓	x	✓	?	?	✓	✓	✓	✓	✓	✓	✓	✓	✓	✓	✓	✓	60- 79%	Low
*Demmler at al., 2018	✓	✓	x	✓	x	✓	?	✓	✓	✓	✓	✓	✓	✓	✓	?	?	✓	?	Low
*Hall et al., 2022	✓	✓	N/A	?	?	?	✓	✓	✓	✓	✓	✓	✓	✓	✓	✓	N/A	?	?	Low

*Panel data studies
